# Clinical features of COVID-19 for integration of COVID-19 into influenza surveillance: A systematic review

**DOI:** 10.7189/jogh.12.05012

**Published:** 2022-04-14

**Authors:** Bohee Lee, Thulani Ashcroft, Eldad Agyei-Manu, Emma Farfan de los Godos, Amanda Leow, Prerna Krishan, Durga Kulkarni, Madhurima Nundy, Karen Hartnup, Ting Shi, Emilie McSwiggan, Harish Nair, Evropi Theodoratou, Ruth McQuillan, the Usher Network for COVID-19 Evidence Reviews (UNCOVER) group

**Affiliations:** 1 Centre for Population Health Sciences, Usher Institute, University of Edinburgh, Edinburgh, Scotland, UK; 2 Asthma UK Centre for Applied Research, University of Edinburgh, Edinburgh, Scotland UK; 3 Centre for Global Health, Usher Institute, University of Edinburgh, Edinburgh, Scotland, UK; 4 Cancer Research UK Edinburgh Centre, MRC Institute of Genetics and Cancer, University of Edinburgh, Edinburgh, Scotland, UK

## Abstract

**Background:**

In November 2020, the World Health Organization (WHO) created interim guidance on how to integrate testing for SARS-CoV-2 into existing influenza surveillance systems. Influenza-like illness (ILI) and severe acute respiratory illness (SARI) case definitions have been used to specify the case definition of COVID-19 for surveillance purposes. This review aims to assess whether the common clinical features of COVID-19 have changed to the point that the criteria used to identify both COVID-19 and influenza in surveillance programs needs to be altered.

**Methods:**

A systematic review of reviews following PRISMA-P guidelines was conducted using the “COVID-19 evidence review” database from August 19, 2020, to August 19, 2021. Reviews providing pooled estimates of the prevalence of clinical features of COVID-19 within the general population, diagnosed by polymerase chain reaction or rapid diagnostic test, were included. These were critically appraised and sensitivity analysis was undertaken to examine potential causes of bias.

**Results:**

Fourteen reviews were identified, including three on adults only and three on children only. For all reviews, combined fever (median prevalence = 73.0%, IQR = 58.3-78.7) and cough (45.1%, IQR = 28.9-54.0) were the most common features. These were followed by loss of taste or smell (45.1%, IQR = 28.9-54.0), hypoxemia (33%, one review), fatigue (26.4%, IQR = 9.0-39.4) and expectoration (23.9%, IQR = 23.3-25.5). Fever and cough continued to be the most prevalent features for adults and children, with subsequent symptoms being similar for adults only. However, the pattern differed for children, with headache (34.3%, IQR = 18-50.7) and nasal congestion (20%, one review) being the third and fourth commonest symptoms.

**Conclusions:**

The prevalent features found in this recent review were the same as the ones identified at the beginning of the pandemic. Therefore, the current approach of using the ILI and SARI criteria which incorporate fever and cough will identify COVID-19 cases in addition to influenza. Interestingly, children may present with different features, as headaches and nasal congestion were more common in this group. Future research could examine this further and investigate whether symptomology changes with new variants of COVID-19.

Since 1952, the World Health Organisation (WHO) has coordinated the global epidemiological and virological surveillance of influenza. The WHO’s Global Influenza Surveillance and Response System (GISRS) was established to provide a global network for influenza surveillance information between countries. This allows countries to provide information on seasonality, notify on outbreaks and novel strains of influenza virus, and provide information on strain selection for the annual formulation of the seasonal influenza vaccine. The coronavirus disease (COVID-19) pandemic caused significant disruptions to influenza surveillance systems across the world. As countries grappled with resources to identify SARS-CoV-2 within their populations, the WHO produced interim guidance to aid countries and maintain or restart influenza surveillance as well as monitoring SARS-CoV-2 [1].

Influenza surveillance programmes use all or a select proportion of cases meeting the case definitions, characterized by a set of pre-defined criteria, to identify individuals for collection of clinical specimens with the aim of identifying transmission of influenza in the population. Case definitions are updated over time with the criteria based on the most common clinical features with the greatest predictive value. As of October 2020, in the case of influenza, common case definitions include influenza-like illness (ILI), which is an acute respiratory infection with a measured fever of ≥38°C and cough with onset within the last 10 days [1]. Other case definitions used in influenza surveillance are acute respiratory infection (ARI) and severe acute respiratory infection (SARI) (**Table 1**). The case definitions for COVID-19 have been reviewed with the growing knowledge of the most common and predictive clinical features. The WHO interim guidance on the integration of COVID-19 into influenza surveillance found the most common clinical features of COVID-19 were fever (83%) and cough (60%), followed by loss of taste/smell (41%), fatigue (31%), and loss of appetite (30%) [1]. These symptoms were included in the current ILI, ARI, and SARI case definitions, and because of this, WHO recommends the continued use of these case definitions with multiplex PCR assays to test respiratory samples for SARS-Cov-2, as well as influenza.

**Table 1:** Case definitions for influenza from WHO global influenza programme interim guidance [1]

Case definition	Abbreviation	Criteria
**Influenza-like illness**	ILI	Symptom onset within past 10 d AND measured fever ≥38°C AND respiratory infection (cough)
Acute respiratory illness*	ARI	At least one of cough, sore throat, shortness of breath, runny nose with or without fever AND a clinician’s judgement that the illness is due to an infection
**Severe acute respiratory illness**	SARI	Severe illness requiring hospitalisation AND symptom onset within past 10 d AND fever reported or measured ≥38°C AND respiratory infection such as cough

ILI – influenza-like illness, ARI – acute respiratory infection

*ARI case definition is used in some countries for influenza and other respiratory virus surveillance [2].

A year later, we have learned more about COVID-19 symptomology, and it is necessary to review guidance for the incorporation of SARS-CoV-2 into influenza surveillance. In that context, it is important to examine whether clinical features of COVID-19 have changed and whether the existing case definitions used for influenza surveillance are still valid for use in identifying persons with COVID-19. We were commissioned by WHO in August 2021 to review the current evidence and to help with updating their guidance on the integration of SARS-CoV-2 into influenza surveillance. Therefore, we systematically reviewed systematic reviews on the clinical features of COVID-19, aiming to identify common clinical features in persons with COVID-19 by age group.

## METHODS

### Data sources

Systematic reviews on the clinical features of COVID-19 were identified from the “COVID-19 evidence review” website supported by the US Department of Veterans Affairs Evidence Synthesis Program in the USA [3]. This website provides completed reviews and reviews in progress from multiple data sources, including the WHO COVID database, Cochrane, and Centres for Evidence-based Medicine Oxford COVID-19 Evidence Service. A search strategy was not required as this database indexes articles using the term “clinical characteristics”. The entry date of each record was extracted using the website “Data miner” (https://dataminer.io/) [4]. We uploaded the retrieved records to web-based software, Covidence, for screening [5].

### Eligibility criteria

The WHO interim guidance included a literature search from the start of the pandemic to August 2020 [1]. Therefore, we searched for reviews published from 19th August 2020 to 19th August 2021. We used Preferred Reporting Items for Systematic Review and Meta-Analysis Protocols (PRISMA-P) to systemically review the literature.

We included systematic reviews reporting the pooled estimate of the prevalence of clinical features of polymerase chain reaction (PCR), or rapid diagnostic test (RDT) confirmed COVID-19 infection seen in hospitals or community settings. Any specific clinical feature was included, but features that were classified using generalised terms for a collection of symptoms were excluded. We also excluded reviews not reporting pooled estimates for clinical features or those not limiting study populations to PCR- or RDT-confirmed COVID-19 cases. Details of eligibility criteria are summarised in **Table 2**.

**Table 2:** Eligibility criteria

	Inclusion criteria	Exclusion criteria
**Population**	Reviews including PCR- or RDT-confirmed COVID-19 cases.	Reviews not limiting their study population to PCR- or RDT-confirmed COVID-19 cases.
**Exposure/Intervention**	Reviews reporting clinical features of polymerase chain reaction (PCR) or rapid diagnostic test (RDT) confirmed COVID-19 within the general population.	Reviews focussing on sub-groups or patients with special medical conditions (eg, patients with comorbidities, pregnant women, etc.)
**Outcome**	Systematic reviews reporting the pooled estimate of the prevalence of clinical characteristics.	1. Reviews not reporting a pooled estimate for clinical features reporting or those that grouped symptoms together
		2. Reviews that focused on syndromes that may be a sequela of COVID-19 (eg, Multisystem inflammatory syndrome in children (MIS-C), Guillain-Barre syndrome and acute respiratory distress syndrome).
**Article type**	Systematic reviews	Non-systematic or narrative reviews
**Language**	Studies published in English	Studies published in languages other than English

### Risk of bias assessment

Risk of bias was assessed using the Joanna Briggs Institute (JBI) Checklist for Systematic Reviews and Research Synthesis. Quality scores per review were calculated by summing all “yes” responses to the 11 questions, such that 11 was the highest score. We regarded studies with scores greater than 8 as high quality and those below as moderate to low quality.

### Selection and extraction

Two reviewers with clinical and public health backgrounds independently screened the title, abstracts, and full texts of all the identified reviews on Covidence. We resolved any conflicts through discussion with a third reviewer. Two authors developed a data extraction template and pilot tested this on three of the included reviews before finalising the template for the final data extraction by the team. The following information was collected from each review: last date of literature search, age group, clinical features, countries, and settings of primary studies included in the review.

For each clinical feature, we collected the point estimate and the corresponding 95% confidence intervals (CI) of the pooled estimate of prevalence. The number of studies and subjects involved in that estimate was also collected. We extracted the estimates separately by age groups such as adults or children, if specified in the original review. Clinical features that were either non-specific or included multiple features within them (eg, gastrointestinal symptoms) were excluded from data analysis; however, prevalence of asymptomatic cases was included. If data were not provided in the review or supplementary material, we contacted authors to request additional data.

### Data analysis

We calculated the median and interquartile range (IQR) of the point estimates for each symptom for the pooled prevalence reported by the reviews. Medians were used as these are not affected by the distribution of data or the presence of outliers, and we did not have access to the full data sets of included reviews to examine this. Clinical features were ordered by their median prevalence. We analysed all the reviews together and then conducted subgroup analyses of children and adults separately. We did not stratify the population using age cut-offs but followed broad age groups defined by each review. A potential source of bias in systematic reviews of reviews occurs if the same primary studies are included several times within the included reviews. Therefore, we investigated the percentage of overlap of primary studies in the included review. Several of the included reviews did not provide details of the primary studies when reporting prevalence estimates for clinical features. Since it was not possible to look at all primary studies (n = 453) a pragmatic approach was taken to assess overlapping using all primary studies which reported any clinical feature in a review. Two additional sensitivity analyses were undertaken. The first compares the ranking of features obtained in the main analysis to the ranking obtained by selecting only the most recent review (as indicated by the last date of search) for each symptom. The second examines the effect of including only reviews with five or more clinical features on the ranking of symptom prevalence found in the main analysis. All data analyses and visualisation were conducted using R software (version 4.1.1).

## RESULTS

### Characteristics of included reviews

**Figure 1:**
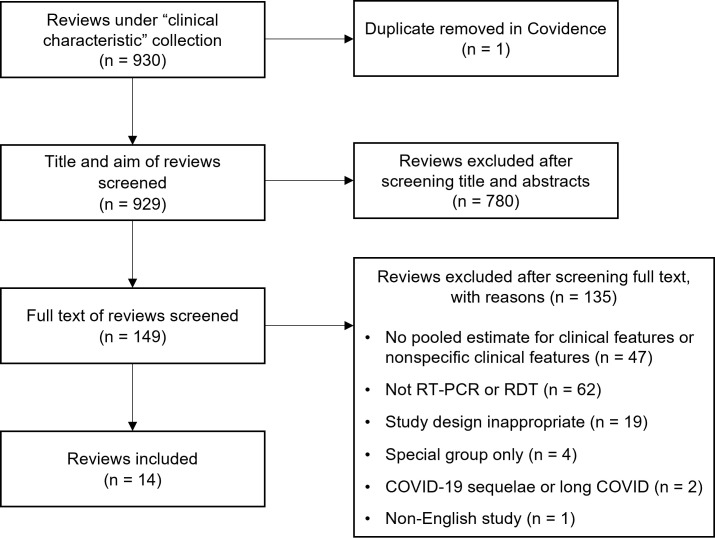
PRISMA flowchart for inclusion of studies.

Of the 930 reviews identified, 14 were included for data extraction [6-19]. **Figure 1** shows the process of selecting eligible studies. The most common exclusion criterion was no information on the method of confirmation of COVID-19. The reviews provided global coverage of primary studies; however, most were from China (69.7%), Europe (23.9%) and the USA (9.0%). When reviews provided information on settings (n = 10), cohorts were predominately hospitalised patients (75.9%) with a smaller number identified from community clinics which included outpatient clinics (9.3%). Twenty-four clinical features were documented for all reviews, 13 were reported in the studies on children only (n = 3 [8,9,11]) and 20 on adults only (n = 3 [10,14,19]). It should be noted that the maximum ages of the three reviews focusing on children were 18 [11], 19 [9] and 21 years old [8], and as such, young adults may be included in these populations. However median ages when provided were 5 years [9] and 8 years [8], suggesting their samples were mostly children. Of the reviews focusing only on adults, one was concerned with those in an aged care facility (median age = 81.5 years) [14]. The other adult-only review included studies with a median age of 48.8 years [19] and a mean age of 61.9 years [10]. Often the review provided characteristics for the whole review rather than only those primary studies providing information on clinical features. Details of characteristics of primary studies included in reviews are available in tables S1 and S2 in the **Online Supplementary Document**.

### Quality of included reviews

A score of eight or greater using the JBI tool was taken as high-quality and a score of seven or lower as moderate to low. Six out of the 14 reviews were considered of high [8,13-15,17,18] and eight moderate to low quality [6,7,9-12,16,19] (see Table S3 in the **Online Supplementary Document**).

### Prevalence of symptoms

**Figure 2:**
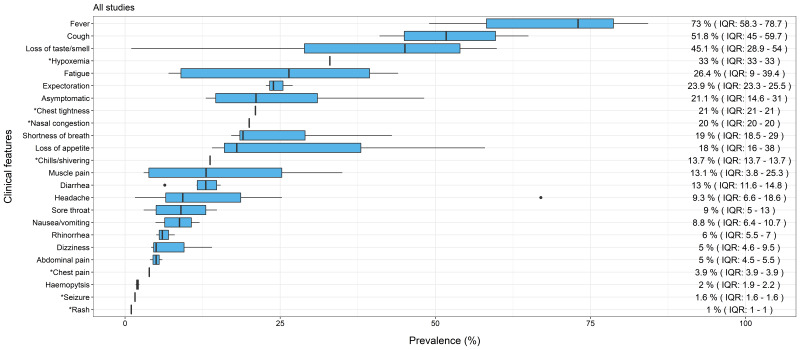
Median prevalence and Interquartile Range (IQR) of clinical features of COVID-19 in all studies. *Single review.

When COVID-19 symptoms for all reviews (all ages) were ranked by order of prevalence, fever was the most prevalent symptom (median = 73.0%, IQR = 58.3-78.7), followed by cough (median = 51.8%, IQR = 45.0-59.7) and then loss of taste or smell (median = 45.1%, IQR = 28.9-54.0). Respiratory symptoms were also common with hypoxemia (median = 33.0% from single review), expectoration (median = 23.9%, IQR = 23.3-25.5) and chest tightness (median = 21% from single review) having prevalence rates above 20%. Fatigue, which is often seen in viral infections, was one of the top five common symptoms, accounting for a median prevalence of 26.4% (IQR = 9.0-39.4). **Figure 2** shows all 24 clinical features of COVID-19 reported with median prevalence and IQR, noting that seven features were obtained from single reviews (see detailed descriptions in Table S4 in the **Online Supplementary Document**).

**Figure 3:**
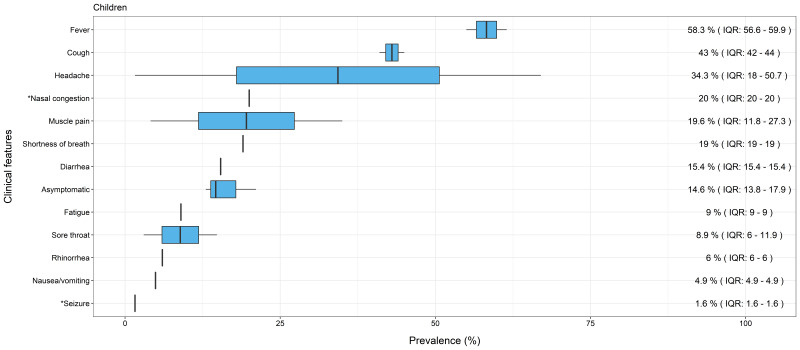
Median prevalence and Interquartile Range (IQR) of clinical features of COVID-19 in children. *Single review.

**Figure 4:**
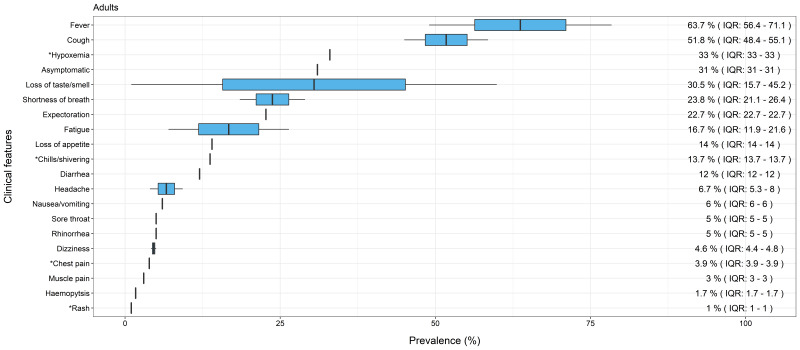
Median prevalence and Interquartile Range (IQR) of clinical features of COVID-19 in adults. *Single review.

Consistent with the analysis of all reviews combined, the most common symptoms reported in reviews focusing only on children (n = 3) were fever (median = 58.3%, IQR = 56.6-59.9) and cough (median = 43%, IQR = 42.0-44.0) (see **Figure 3**). Similarly, the pattern observed in reviews focusing only on adults (n = 3) also showed fever (median = 63.7%, IQR = 56.4-71.1), and cough (median = 51.8%, IQR = 48.4-55.1) (see **Figure 4**) as the most prevalent features. Symptoms in children then appeared to diverge from those in all ages combined and in adults only. For children, headache (median = 34.3%, IQR = 18-50.7), nasal congestion (median = 20% from single review) and muscle pain (median = 19.6%, IQR = 11.8-27.3) were within the top five features. In comparison, common features for adults only were similar to all reviews combined, with loss of taste and smell (median = 30.5%, IQR = 15.7-45.2) and hypoxemia (prevalence = 33% from a single review) present within the top five symptoms of both. There was a lower prevalence of headache in all reviews combined (median = 9.3%, IQR = 6.6-18.6) and adults only (median = 6.7%, IQR = 5.3-8.0), and nasal congestion was only reported in a single systematic review for children [8]. Lastly, asymptomatic cases were also reported by reviews. Combining all reviews, the median prevalence was 21.1% (IQR = 14.6-31.0) and a single review for adults alone found the prevalence to be 31% [14]. Interestingly, it was lower for children only, at 14.6% (IQR = 13.8-17.9); however, none of the reviews differentiated pre-symptomatic and asymptomatic cases. Therefore, we are not able to comment if those classified as asymptomatic went on to develop symptoms.

### Sensitivity analyses

**Figure 5:**
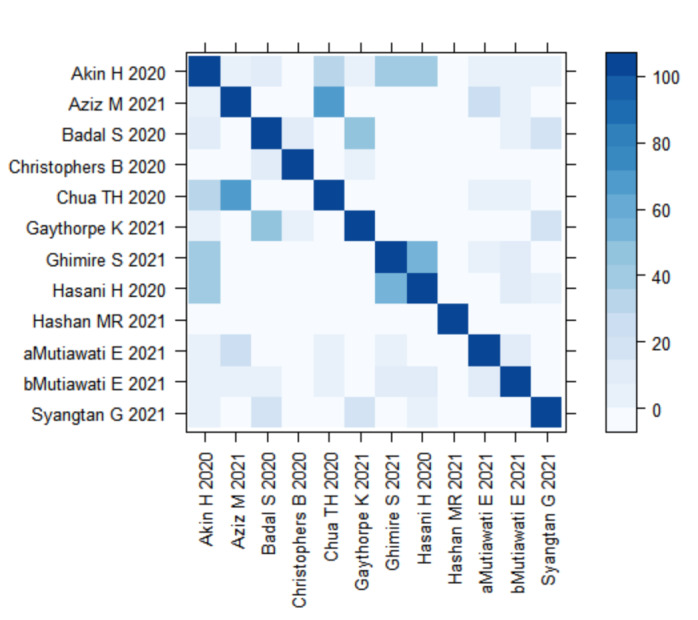
Heatmap of overlapping studies. *Degree of shading shows amount of overlap, such that dark blue is 100% and light blue is 0% overlap.

The degree of overlap of primary studies included in the reviews is shown in **Figure 5**. The number of overlapping studies was found to be low in all but two reviews. Mutiawati et al. [16] and Aziz et al. [7] both examined loss of taste and smell and were found to be moderately overlapped, with 27 studies being present in both. When Aziz et al [7] was excluded (as it contained a smaller number of studies) the overall median prevalence of loss of taste and smell decreased from 45.1% to 38.2% without changing the ranking of clinical features in all reviews combined. Lists of primary studies and numbers of overlapping studies per review are shown in tables S5 and S6 in the **Online Supplementary Document**.

The additional sensitivity analyses based on reviews with at least five different clinical features and using the most recent review only are shown in Figures S1 to S6 in the **Online Supplementary Document**. When examining reviews that reported more than five clinical features, we found seven reviews for all ages, and two reviews each on children only and adults only.

There was little change in the prevalence of symptoms for these reviews, except that loss of taste and/or smell became the lowest ranking. This is not surprising, given that two reviews were excluded that were solely focused on this symptom [7,16]. It remained lowest when most recent reviews were examined on adults only (n = 2) but returned to 3rd ranking features for all ages combined (n = 8). This suggests that loss of taste and smell remains a prominent feature for those with COVID-19. Another finding from examining the most recent review in children (n = 3) was that headache became the commonest feature. with a median prevalence of 67%. It was followed by fever and cough, whereas for adults only and when all reviews were combined fever and cough continued to be the most prevalent clinical feature.

## DISCUSSION

Our findings show that fever was the most prevalent symptom, closely followed by cough for adults and children. The subsequent features – shortness of breath, hypoxemia, and expectoration – are common symptoms of respiratory tract infections. These trends are in line with a recent report by the International Severe Acute Respiratory and Emerging Infections Consortium (ISARIC) report on 60 109 hospitalised cases of COVID-19 across 43 countries [20]. According to this report, the three most common symptoms at admission were history of fever (68.7% of patients), cough (68.5%), and/or shortness of breath (65.8%), with 92% of those admitted experiencing one or more of these features. The analysis by ISARIC found additional symptoms similar to those in our report, but with a higher prevalence. For example, they found fatigue in 46.4%, whilst our systematic review found the prevalence to be 26.4% (IQR = 9.0-39.4). Fatigue was within the top five features for our review; however, the estimated prevalence for the less common symptoms (eg, diarrhoea, nausea and vomiting, headache, sore throat) was lower. Thus, it followed the same pattern as the ISARIC analysis, for most features apart from the loss of taste and smell. The striking lower incidence of loss of taste (7.2%) or smell (6.2%) at admission in the ISARIC report is at odds with our review (45.1%, IQR = 28.9-54.0). Our review combined the estimate of loss of taste and/or smell rather than reporting them separately. Though symptoms were derived predominantly from hospitalised patients in both cases, our review included mainly studies in China, whilst 79% of the cohort in ISARIC were from the United Kingdom. There will be differences in health care systems and health care-seeking behaviours of these populations that may explain this difference. A study using self-reported symptoms of COVID-19 via six digital platforms in the UK, USA, and Israel found loss of taste and smell to be the most consistent symptoms associated with a positive SARS-CoV-2 test [21]. The cohorts included were from a community with possibly milder symptoms. However, it highlights the importance of loss of taste and/or smell as a feature of COVID-19 at this time, and it is currently included within the criteria for testing for SARS-CoV-2 in the United Kingdom and in the USA.

A further remarkable finding from our report was the different symptomology in children compared to adults and all ages, with headache, muscle pain and nasal congestion being more prominent features in children. A prospective cohort study in the UK of symptomatic children (5-17 years) using a digital platform for self-reporting symptoms found headache and fatigue [22] to be the most common symptoms. These children were community cases. A systematic review of systematic reviews in children only found fever and cough to be the most prevalent symptom [23] in hospitalised children. These findings from both community and hospitalised cases are consistent with our review, which found fever, cough, headache, nasal congestion, and muscle pain to be the five most common features of COVID-19 in children. We note that understanding symptomology in children is difficult as it is mainly elicited from the caregiver and therefore may not be an accurate representation of the symptoms of the child.

### Strengths and limitations

Our review has several strengths. To the best of our knowledge, this is the first study covering a wide range of clinical features of COVID-19. The articles included in our review had global coverage and included all populations and ages. Our findings that fever, cough and other respiratory symptoms continue to be the most common features of COVID-19 suggested that continued use of ILI, ARI and SARI as case definitions will be sufficient to identify both influenza and SARS-CoV-2 for surveillance purposes. In addition, we examined bias that may arise through the inclusion of a review that focused on specific features by sensitivity analyses. These reviews may introduce publication bias by suggesting these features are more common than those reporting all or multiple features.

Nonetheless, our study is not free from limitations. Although we undertook sensitivity analysis to investigate overlapping primary studies, it might not be possible to completely resolve the double-counting issue of clinical features. We attempted to examine the overlap of clinical features; however, it was not possible to extract a list of primary studies per clinical feature. Even the pragmatic approach we took by extracting all primary studies accounting for all clinical features, excluded two reviews that did not provide a list of primary studies [15,19]. Several reviews did not report the sample size per clinical feature, which prevented the calculation of other estimates such as means or weighted estimates. We acknowledge potential errors, as we did not recalculate the pooled estimates using primary studies and only provided medians for pooled estimates. Additionally, we note that some reviews did not provide data on the settings, and we surmise that the majority were hospital admissions so those with milder illnesses might not be represented in this review.

Our review may have missed some information on clinical features. We excluded some reviews due to their unclear eligibility criteria. For instance, studies not specifically mentioning RT-PCR or RDT tests were excluded from this review. Therefore, it is possible that we missed some reviews or primary studies using RT-PCR or RDT tests. Furthermore, our review might miss some information because some reviews were selective and only extracted data on certain features from the included primary studies. This may can explain the small number of reviews for children or adults alone and that seven clinical features were provided from single reviews.

Only six out of 14 reviews were considered high-quality, and several limitations of this review are related to the lack of information provided by included reviews. As we used the same weight for all items on the JBI critical appraisal tool, some significant items for this review might be underestimated. In addition, we were not able to appraise the quality of the primary studies in the included reviews, which is another potential source of error. Future reviews need to improve methodological rigour, consider applying different weights to items for quality appraisal, and examine overlap at a primary study level. Although we rigorously searched the literature, primary studies published since the latest search date of the included reviews (November 2020) will be missed, and future studies should consider including reviews or primary studies published from this date.

The predominant symptoms found by the interim WHO guidance [1] (fever and cough) were identical to those found by our report. There has been evidence to suggest certain variants of SARS-CoV-2 may have different symptomology. Genomic sequencing is only undertaken in a small subset of positive SARS-CoV-2 samples and the ability of countries to conduct testing varies greatly. Though the literature we reviewed did not specify variants, it can be speculated that common clinical features of COVID-19 at specific time periods were attributable to the predominant circulating variant. This will vary by country as well as by time. The Office of National Statistics (ONS) in the UK have compared symptoms recorded for Delta (B.1.617.2) and Omicron (B.1.1.529) variants in December 2021. Findings suggest that whilst fever, cough, shortness of breath and other features of viral illnesses (eg, fatigue, weakness, myalgia) are similar, loss of taste and smell were more common with the Delta variant [24]. There may be more reports of sore throat with Omicron, but the report also found an increase in sore throat symptoms in those that tested negative [24]. Given the rapid emergence of new variants, further studies should consider changes of clinical features with the predominance of certain variants. Although GIRS programs have begun to be re-established in many parts of the world, with more countries using multiplex PCR testing on respiratory samples, it is critical to assess whether these case definitions are successful in capturing both influenza and SARS-CoV-2.

## CONCLUSION

Fever and cough were the most common clinical features for all ages and for adults and children separately. Other prevalent features for all ages and adults included loss of taste or smell, fatigue, shortness of breath, hypoxia, and expectoration. Children may present with different features, as headache and nasal congestion were more common in this group. These results are in line with current evidence, suggesting that surveillance case definitions used for ILI, ARI and SARI are likely to capture SARS-CoV-2 and can be continued to be used for influenza surveillance.

## Additional material


Online Supplementary Document
