# Supplementary material for: Clinical features of COVID-19 for integration of COVID-19 into influenza surveillance: A systematic review
Source: J Glob Health. 2022 Apr 14;12:05012. doi: 10.7189/jogh.12.05012 (PMC9107308; doi:10.7189/jogh.12.05012)

Online Supplementary Document

Table S1. Summary characteristics of primary studies from reviews which provided data for pooled estimates

Included review	Number of primary studies	Age (yrs.)[§]	Male (%)	Countries, n (%)	Setting*, n (%)	Number of clinical features[$]
**Akin, 2020**	76	**All ages**<br>NP	NP	China 72 (94.7)<br>USA 8 (10.5)<br>USA & Canada 1 (1.3)<br>Australia 1 (1.3)<br>Europe 10 (13.2)<br>Unknown 1 (1)	Hospital 45 (59)<br>Community 4 (5)<br>Both 27 (36)	4
**Aziz, 2021**	51	**All ages**<br>Mean 46.7 (SD 10.4)	46.9	China 2 (3.9)<br>USA 5 (9.8)<br>Canada 2 (3.9)<br>Europe 31 (60.8)<br>China & Europe 1 (2.0)<br>Asia 4 (7.8)<br>Turkey 4 (7.8)<br>Iran 2 (3.9)	Hospital 42 (82)<br>Community 9 (18)	1
**Badal, 2020[†]**	20	**Children**<br>8 (0 -21)	NP<br>Ratio M: F 1:1.34	China 13 (65.0)<br>USA 3 (15.0)<br>USA & Canada 1 (5.0)<br>Europe 3 (15.0)	NP	10
**Christophers, 2020**	22	**Children**<br>5 (0 – 19)	54.5	China 100 (81.3)<br>USA 2 (1.6)<br>Europe 14 (11.4)<br>Others 7 (5.7)	Hospital 22 (100)	9
**Chua, 2020**	3	**Adults**<br>NP	NP	China 1 (33.3)<br>Europe 2 (66.6)	Hospital 1 (33)<br>Community 1 (33)<br>Both 1 (33)	1

**Gaythorpe, 2021**	14	**Children**<br>0 - 18	NP	China 11 (79)<br>Europe 1 (7)<br>Turkey 1 (7)<br>Others 1 (7)	Hospital 7 (50)<br>Community 2 (14)<br>Unknown 5 (36)	1
**Ghimire, 2021**	38	**All ages**<br>NP	NP	China 27 (71)<br>USA 5 (13)<br>Europe 3 (8)<br>Asia 2 (5)<br>Mexico 1 (3)	Hospital 38 (100)	2
**Hasani, 2020**	14	**All ages**<br>0 - 94	NP	China 14 (100)	Hospital 12 (85.7)<br>Community 1 (7.1)<br>Both 1 (7.1)	10
**Hashan, 2021**	14	**Adults**<br>NP	44	China 1 (7.1)<br>USA 5 (35.7)<br>Europe 8 (57.1)	Hospital 14 (100)	16
**Kim, 2021[†]**	80	**All ages**<br>Mean 68.5	57	China 43 (53.8)<br>USA 10 (12.5)<br>Europe 22 (24.4)<br>Others 5 (6.3)	NP	16
**[a]Mutiawati, 2021**	107	**All ages**<br>NP	NP	China 4 (3.7)<br>USA 13 (12.1)<br>UK 3 (2.8)<br>Canada 2 (1.9)<br>Europe 41 (38.3)<br>Others 43 (40.2)	NP	1 (loss of taste/smell)
**[b]Mutiawati, 2021**	78	**All ages**<br>NP	NP	China 47 (60.3)<br>USA 5 (6.4)<br>Europe 14 (17.9)<br>Other 12 (15.4)	Hospital 58 (74.4)<br>Community 8 (10.3)<br>Both 5 (8.6)<br>Unknown 5 (8.6)	1 (headache)
**Syangtan, 2021**	16	**All ages**<br>≤ 18: 42.5%,	54.4	China 11 (68.8)<br>Japan 2 (12.5)	Hospital 7 (43.8)	1

		19–50: 22.8%<br>≥51: 34.7%		USA 3 (18.8)	Community 5 (31.3)<br>Unknown 4 (25)	
**Xie, 2021**[†]	90	**Adults**<br>48.8 (37 – 68)	53.1	China 88 (97.8)<br>Australia 1 (1.1)<br>Singapore 1 (1.1)	NP	10

§ Data are median (Range) or n (%), unless indicated otherwise; NP = not provided, SD= standard deviation; *community includes outpatient clinics; $only clinical features that were extracted for this review (some reviews included non-specific or combinations of features which is not included in this number; † reviews which did not provide details of primary studies used for each clinical feature;

Table S2: Detailed findings from individual reviews on clinical features

Authors	Age group	Clinical features	No. of study	No. of patients	Point estimate	Lower bound of 95% CI	Upper bound of 95% CI
Akin H, 2020[5]	all	Fever	45	74543	73	70	76
Akin H, 2020[5]	all	Abdominal pain	38	34713	6	4	7
Akin H, 2020[5]	all	Loss of appetite	37	22743	18	10	27
Akin H, 2020[5]	all	Diarrhoea	44	62892	15	12	19
Akin H, 2020[5]	all	Nausea/vomiting	44	46390	10	8	12
Aziz M, 2021[6]	all	Loss of taste/smell	51	11074	52	42.5	61.6
Badal S, 2020[7]	children	Asymptomatic	20	222	13	11	14
Badal S, 2020[7]	children	Fever	19	592	55	52	58
Badal S, 2020[7]	children	Cough	17	467	45	42	49
Badal S, 2020[7]	children	Shortness of breath	11	169	19	16	22
Badal S, 2020[7]	children	Headache	5	117	67	60	74
Badal S, 2020[7]	children	Rhinorrhoea	9	38	6	4	8
Badal S, 202[7]	children	Nasal congestion	8	69	20	16	25
Badal S, 2020[7]	children	Sore throat	11	18	3	2	4
Badal S, 2020[7]	children	Muscle pain	7	194	35	32	40
Badal S, 2020[7]	children	Fatigue	6	53	9	7	11
Christophers B, 2020[8]	children	Asymptomatic	22	18	14.6	NA	NA
Christophers B, 2020[8]	children	Fever	22	75	61.5	NA	NA
Christophers B, 2020[8]	children	Cough	22	50	41	NA	NA
Christophers B, 2020[8]	children	Headache	22	2	1.6	NA	NA
Christophers B, 2020[8]	children	Sore throat	22	18	14.8	NA	NA
Christophers B, 2020[8]	children	Diarrhoea	22	19	15.4	NA	NA
Christophers B, 2020[8]	children	Nausea/vomiting	22	6	4.9	NA	NA
Christophers B, 2020[8]	children	Muscle pain	22	5	4.1	NA	NA
Christophers B, 2020[8]	children	Seizure	22	2	1.6	NA	NA
Chua TH, 2020[9]	adults	Loss of taste/smell	3	703	59.9		
Gaythorpe K, 2021[10]	children	Asymptomatic	14		21.1	14	28.1
Ghimire S, 2021[11]	all	Diarrhoea	37	8352	11.52	8.97	14.68
Ghimire S, 2021[11]	all	Nausea/vomiting	26	7196	7.53	5.27	10.65
Hasani H, 2020[12]	all	Fever	14		84.3	78.6	88.7
Hasani H, 2020[12]	all	Cough	14		60.1	53.5	66.4
Hasani H, 2020[12]	all	Expectoration	5		23.9	16.4	33.4
Hasani H, 2020[12]	all	Shortness of breath	8		17.1	9.1	29.8
Hasani H, 2020[12]	all	Haemoptysis	5		2.3	0.9	5.7
Hasani H, 2020[12]	all	Headache	12		9.1	7	11.8

Hasani H, 2020[12]	all	Sore throat	5		13	8.5	19.3
Hasani H, 2020[12]	all	Diarrhoea	11		6.4	4.3	9.5
Hasani H, 2020[12]	all	Nausea/vomiting	2		10.9	3.8	27.5
Hasani H, 2020[12]	all	Fatigue	11		39.4	29.1	50.8
Hashan MR, 2021[13]	adults	Asymptomatic	13	985	31	28	34
Hashan MR, 2021[13]	adults	Fever	13	1872	49	47	52
Hashan MR, 2021[13]	adults	Cough	14	1876	45	43	47
Hashan MR, 2021[13]	adults	Shortness of breath	10	1497	29	27	32
Hashan MR, 2021[13]	adults	Dizziness	2	89	5	2	12
Hashan MR, 2021[13]	adults	Headache	7	1116	4	3	5
Hashan MR, 2021[13]	adults	Loss of taste/smell	2	313	1	0	1
Hashan MR, 2021[13]	adults	Rhinorrhoea	5	1331	5	4	6
Hashan MR, 2021[13]	adults	Sore throat	6	1128	5	4	7
Hashan MR, 2021[13]	adults	Loss of appetite	9	1712	14	13	16
Hashan MR, 2021[13]	adults	Diarrhoea	7	647	12	10	15
Hashan MR, 2021[13]	adults	Nausea/vomiting	6	528	6	4	8
Hashan MR, 2021[13]	adults	Muscle pain	5	256	3	1	6
Hashan MR, 2021[13]	adults	Fatigue	5	1162	7	6	9
Hashan MR, 2021[13]	adults	Hypoxia	5	373	33	29	38
Hashan MR, 2021[13]	adults	Rash	1	103	1	0	7
Kim H, 2021[14]	all	Fever	32	NA	79	70	86
Kim H, 2021[14]	all	Cough	31	NA	65	60	70
Kim H, 2021[14]	all	Expectoration	16	NA	27	20	35
Kim H, 2021[14]	all	Shortness of breath	28	NA	43	34	52
Kim H, 2021[14]	all	Dizziness	5	NA	14	4	28
Kim H, 2021[14]	all	Headache	19	NA	12	8	16
Kim H, 2021[14]	all	Rhinorrhoea	5	NA	8	4	12
Kim H, 2021[14]	all	Sore throat	15	NA	9	6	12
Kim H, 2021[14]	all	Abdominal pain	5	NA	4	1	9
Kim H, 2021[14]	all	Loss of appetite	8	NA	58	43	72
Kim H, 2021[14]	all	Diarrhoea	25	NA	14	10	19
Kim H, 2021[14]	all	Nausea/vomiting	12	NA	12	7	17
Kim H, 2021[14]	all	Nausea/vomiting	7	NA	8	2	18
Kim H, 2021[14]	all	Muscle pain	22	NA	22	17	27
Kim H, 2021[14]	all	Fatigue	17	NA	44	32	55
Kim H, 2021[14]	all	Chest tightness	10	NA	21	11	33
Mutiawati E, 2021[15]	all	Loss of taste/smell	107	32142	38.2	36.5	47.2
Mutiawati E, 2021[16]	all	Headache	78	104751	25.26	NA	NA
Syangtan G, 2021[27]	all	Asymptomatic	16	2788	48.2	30	60
Xie J, 2021[24]	adults	Fever	65	10881	78.4	74.5	82.3
Xie J, 2021[24]	adults	Chills/Shivering	14	3841	13.7	8.1	19.2
Xie J, 2021[24]	adults	Chest pain	17	3197	3.9	2.4	5.4
Xie J, 2021[24]	adults	Cough	62	10571	58.5	51.4	65.6

Xie J, 2021[24]	adults	Expectoration	35	6964	22.7	18.2	27.2
Xie J, 2021[24]	adults	Shortness of breath	19	5964	18.5	12.9	24.2
Xie J, 2021[24]	adults	Haemoptysis	6	2039	1.7	0.7	2.8
Xie J, 2021[24]	adults	Dizziness	10	1831	4.2	2.5	5.9
Xie J, 2021[24]	adults	Headache	35	7379	9.3	7.6	11
Xie J, 2021[24]	adults	Fatigue	45	8938	26.4	21.4	31.4

*NA: Not available

Table S3: Quality assessment

Author (year)	1. Is the review question clearly and explicitly stated?	2. Were the inclusion criteria appropriate for the review question?	3. Was the search strategy appropriate?	4. Were the sources and resources used to search for studies adequate?	5. Were the criteria for appraising studies appropriate?	6. Was critical appraisal conducted by two or more reviewers independently?	7. Were there methods to minimize errors in data extraction?	8. Were the methods used to combine studies appropriate?	9. Was the likelihood of publication bias assessed?	10. Were recommendations for policy and/or practice supported by the reported data?	11. Were the specific directives for new research appropriate?	Total (yes)
**Akin,2020**	Yes	Yes	Yes	Yes	No	No	No	Yes	No	Yes	Yes	7
**Aziz M 2021**	Yes	Yes	No	Yes	Yes	No	No	Yes	Yes	No	Yes	7
**Badal S 2020**	Yes	Yes	Yes	Yes	Yes	Yes	Yes	Yes	Yes	Yes	Yes	11
**Christophers B 2020**	Yes	Yes	No	Yes	Yes	Yes	Yes	No	No	Yes	No	7
**Chua TH 2020**	Yes	Yes	No	No	No	No	No	Yes	No	No	No	3
**Gaythorpe 2021**	Yes	Yes	No	No	Yes	Yes	No	Yes	Yes	No	No	6
**Ghimire S 2021**	Yes	Yes	Yes	No	No	Yes	Yes	Yes	Yes	No	No	7
**Hasani H 2020**	Yes	Yes	Yes	Yes	Yes	No	Yes	Yes	Yes	No	Yes	9
**Hashan MR 2021**	Yes	Yes	Yes	Yes	Yes	Yes	Yes	Yes	Yes	Yes	Yes	11
**Kim H 2021**	Yes	Yes	Yes	Yes	Yes	Yes	No	Yes	Yes	No	Yes	9
**[a]Mutiawati E 2021**	Yes	Yes	Yes	Yes	Yes	No	No	Yes	Yes	No	No	7
**[b]Mutiawati E 2021**	Yes	Yes	Yes	Yes	Yes	Yes	Yes	Yes	Yes	Yes	Yes	11
**Syangtan G 2021**	Yes	Yes	Yes	Yes	Yes	Yes	No	Yes	Yes	Yes	NA	9
**Xie J 2021**	Yes	Yes	No	Yes	Unclear	Yes	Yes	Yes	Yes	No	Unclear	7

Table S4: Summary of clinical features reported in reviews ordered by system

Lead author, year	Last date of search	Country	Age group	Severity	Data availability for pooled estimate of symptom prevalence																									
					Asymptomatic	Fever	Chills/shivering	Cough	Expectoration	Shortness of breath	Chest pain	Chest tightness	Haemoptysis	Hypoxemia	Palpitation	Dizziness	Headache	Impaired consciousness	Seizures	Loss of taste/smell	Rhinorrhoea	Nasal congestion	Sore throat	Abdominal pain	Loss of appetite	Diarrhoea	Nausea/vomiting	Myalgia	Fatigue	Rash
*Akin H, 2020[5]	19-09-2020	Global	All ages	All		✓																		✓	✓	✓	✓			
Aziz M, 2021[6]	06-08-2020	Global	All ages	All																✓										
*Badal S, 2020[7]	16-06-2020	Global	Children	All	✓	✓		✓		✓							✓				✓	✓	✓					✓	✓	
*Christophers B, 2020[8]	15-05-2020	Global	Children	All	✓	✓		✓									✓		✓				✓			✓	✓	✓		
Chua TH, 2020[9]	11-05-2020	Global	Adults	All																✓										
Gaythorpe K, 2021[10]	04-07-2020	Global	Children	All	✓																									
*Ghimire S, 2021[11]	05-2020	Global	All ages	All																						✓	✓			
Hasani H, 2020[12]	25-03-2020	China	All ages	All		✓		✓	✓	✓			✓				✓						✓			✓	✓		✓	
*Hashan MR 2021[13]	28-09-2020	Global	Adults	All	✓	✓		✓		✓				✓		✓	✓			✓	✓		✓		✓	✓	✓	✓	✓	✓
Kim H, 2021[14]	08-06-2020	Global	All ages	All		✓		✓	✓	✓		✓				✓	✓				✓		✓	✓	✓	✓	✓	✓	✓	
Mutiawati E 2021[15]	02-09-2020			All													✓													
Mutiawati E 2021[16]	10-11-2020	Global	All ages	All																✓										

Lead author, year	Last date of search	Country	Age group	Severity	Data availability for pooled estimate of symptom prevalence																									
					**Asymptomatic**	**Fever**	**Chills/shivering**	**Cough**	**Expectoration**	**Shortness of breath**	**Chest pain**	**Chest tightness**	**Haemoptysis**	**Hypoxemia**	**Palpitation**	**Dizziness**	**Headache**	**Impaired consciousness**	**Seizures**	**Loss of taste/smell**	**Rhinorrhoea**	**Nasal congestion**	**Sore throat**	**Abdominal pain**	**Loss of appetite**	**Diarrhoea**	**Nausea/vomiting**	**Myalgia**	**Fatigue**	**Rash**
**Syangtan G 2021**[17]	30-04-2020	Global	All ages	All	✓																									
***Xie J, 2021**[18]	18-03-2020	Asia/ Pacific	Adults	All		✓	✓	✓		✓	✓		✓			✓	✓												✓	
				**Total**	5	7	1	6	2	5	1	1	2	1	0	3	7	0	1	4	3	1	5	2	3	6	6	4	4	1

*Excluded features that were non-specific or multiple features grouped together (the number of reviews where these exclusions apply in brackets): gastrointestinal symptoms (4), respiratory symptoms including upper and lower respiratory symptoms (3), nasal symptoms (2), chest distress (1), weakness (1), myalgia or arthritis as a combined category (1), others as a combined category of several symptoms or signs (2).

Table S5 List of primary studies for included reviews

Included reviews	Primary studies	Title
Akin H 2020	Fanelli V	Acute kidney injury in SARS-CoV-2 infected patients.
Akin H 2020	Docherty AB	Featuresof 16, 749 hospitalised UK patients with COVID-19 using the ISARIC WHO Clinical Characterization Protocol.
Akin H 2020	CDC USA (A Children)	Coronavirus disease 2019 in children
Akin H 2020	CDC USA (B Health care professionals)	Characteristics of Health Care Personnel with COVID-19
Akin H 2020	Borobia A	A Cohort of Patients with COVID-19 in a Major Teaching Hospital in Europe
Akin H 2020	Elmunzer BJ	Digestive Manifestations in Patients Hospitalized with COVID-19
Akin H 2020	Guan WJ (A)	Comorbidity and its impact on 1590 patients with COVID-19 in China: a nationwide analysis
Akin H 2020	Luo S	Don't Overlook Digestive Symptoms in Patients With 2019 Novel Coronavirus Disease (COVID-19).
Akin H 2020	Guan WJ (B)	Clinical Characteristics of Coronavirus Disease 2019 in China
Akin H 2020	Hajifathalian K	Gastrointestinal and Hepatic Manifestations of 2019 Novel Coronavirus Disease in a Large Cohort of Infected Patients From New York: Clinical Implications
Akin H 2020	Gil-Rodrigo A	Analysis of clinical characteristics and outcomes in patients with COVID-19 based on a series of 1000 patients treated in Spanish emergency departments
Akin H 2020	Kuang Y	Epidemiological and clinical characteristics of 944 cases of 2019 novel Coronavirus infection of non-COVID-19 exporting city, Zhejiang, China.
Akin H 2020	Jin X	Epidemiological, clinical and virological characteristics of 74 cases of coronavirus-infected disease 2019 (COVID-19) with gastrointestinal symptoms.
Akin H 2020	COVID-19 National Incident Room Surveillance Team Australia	Epidemiology Report 13 (Reporting week to 23: 59 AEST 26 April 2020). Commun. Dis. Intell. (2018) 2020, 44, 1–27.
Akin H 2020	Livanos AE	Gastrointestinal involvement attenuates COVID-19 severity and mortality.
Akin H 2020	Lui W	Analysis of 2019 Novel Coronavirus Infection and Clinical Characteristics of Outpatients: An Epidemiological Study from the Fever Clinic in Wuhan, China
Akin H 2020	Shu L	Clinical Characteristics of 545 Cases Confirmed COVID-19 in Wuhan Stadium Cabin Hospital.
Akin H 2020	Han Y	Epidemiological Assessment of Imported Coronavirus Disease 2019 (COVID-19) Cases in the Most Affected City Outside of Hubei Province, Wenzhou, China.
Akin H 2020	Wen Y	Epidemiological and clinical characteristics of COVID-19 in Shenzhen, the largest migrant city of China.
Akin H 2020	Leichen JR	Olfactory and gustatory dysfunctions as a clinical presentation of mild-to-moderate forms of the coronavirus disease (COVID-19): A multicenter European study.
Akin H 2020	Bannaga S	C-reactive protein and albumin association with mortality of hospitalised SARS-CoV-2 patients: A tertiary hospital experience.
Akin H 2020	Redd WD	Prevalence and characteristics of gastrointestinal symptoms in patients with severe acute respiratory syndrome coronavirus 2 infection in the United States: A multicenter cohort study.

Included reviews	Primary studies	Title
Akin H 2020	Fang D	Manifestations of digestive system in hospitalized patients with novel coronavirus pneumonia in Wuhan, China: A single-center, descriptive study.
Akin H 2020	CDC COVID-19 USA	Coronavirus Disease 2019 in Children—United States, 12 February–2 April 2020.
Akin H 2020	Chen X	Epidemiological and clinical features of 291 cases with coronavirus disease 2019 in areas adjacent to Hubei, China: A double-center observational study.
Akin H 2020	Nobel YR	Gastrointestinal Symptoms and COVID-19: Case-Control Study from the United States.
Akin H 2020	Chen T	Clinical characteristics of 113 deceased patients with coronavirus disease 2019: Retrospective study.
Akin H 2020	Qi D	Epidemiological and clinical features of 2019-nCoV acute respiratory disease cases in Chongqing municipality, China: A retrospective, descriptive, multiple-center study.
Akin H 2020	Zhou Z	Effect of Gastrointestinal Symptoms in Patients With COVID-19.
Akin H 2020	Chen J	Clinical progression of patients with COVID-19 in Shanghai, China.
Akin H 2020	Lui L	A preliminary study on serological assay for severe acute respiratory syndrome coronavirus 2 (SARS-CoV-2) in 238 admitted hospital patients.
Akin H 2020	Zhang G	Clinical features and short-term outcomes of 221 patients with COVID-19 in Wuhan, China.
Akin H 2020	Chen C	Favipiravir versus arbidol for COVID-19: A randomized clinical trial.
Akin H 2020	Xu Z	Cannot find this paper in references there is a Xu YH a Xu XW a Xu W a Xu X ?? Typo for one of these
Akin H 2020	Zhang G	Clinical features and short-term outcomes of 221 patients with COVID-19 in Wuhan, China.
Akin H 2020	Zhang Y	Gastrointestinal tract symptoms in coronavirus disease 2019: Analysis of clinical symptoms in adult patients.
Akin H 2020	Pan L	Clinical characteristics of COVID-19 patients with digestive symptoms in Hubei, China: A descriptive, cross-sectional, multicenter study.
Akin H 2020	Wei L	Clinical Findings of 100 Mild Cases of COVID-19 in Wuhan: A Descriptive Study.
Akin H 2020	Fu L	Influence factors of death risk among COVID-19 patients in Wuhan, China: A hospital-based case-cohort study
Akin H 2020	Xiaong X	Are COVID-19 infected children with gastrointestinal symptoms different from those without symptoms? A comparative study of the clinical characteristics and epidemiological trend of 244 pediatric cases from Wuhan.
Akin H 2020	Huang M	Epidemiological and Clinical Features of 197 Patients Infected with 2019 Novel Coronavirus in Chongqing, China: A Single Center Descriptive Study.
Akin H 2020	Yan Y	Clinical characteristics and outcomes of patients with severe covid-19 with diabetes.
Akin H 2020	Zhou F	Clinical course and risk factors for mortality of adult inpatients with COVID-19 in Wuhan, China: A retrospective cohort study.
Akin H 2020	Gong J	A tool to early predict severe 2019-novel coronavirus pneumonia (COVID-19): A multicenter study using the risk nomogram in Wuhan and Guangdong, China.
Akin H 2020	Garg S	Risk Factors for COVID-19-associated hospitalization: COVID-19-Associated Hospitalization Surveillance Network and Behavioral Risk Factor Surveillance System
Akin H 2020	Chen D	Hypokalemia and Clinical Implications in Patients with Coronavirus Disease 2019 (COVID-19)
Akin H 2020	Lu X	SARS-CoV-2 infection in children.

Included reviews	Primary studies	Title
Akin H 2020	Garazzino S	Multicentre Italian study of SARS-CoV-2 infection in children and adolescents, preliminary data as at 10 April 2020.
Akin H 2020	Zheng F	Clinical characteristics of 161 cases of corona virus disease 2019 (COVID-19) in Changsha.
Akin H 2020	Mo P	Clinical characteristics of refractory COVID-19 pneumonia in Wuhan, China.
Akin H 2020	Yang W	Clinical characteristics and imaging manifestations of the 2019 novel coronavirus disease (COVID-19): A multi-center study in Wenzhou city, Zhejiang, China.
Akin H 2020	Fan Z	Clinical features of COVID-19-related liver damage.
Akin H 2020	Chen Q	Clinical characteristics of 145 patients with corona virus disease 2019 (COVID-19) in Taizhou, Zhejiang, China.
Akin H 2020	Zhang JJ	Clinical characteristics of 140 patients infected with SARS-CoV-2 in Wuhan, China.
Akin H 2020	Wang D	Clinical characteristics of 138 hospitalized patients with 2019 novel coronavirus–infected pneumonia in Wuhan, China.
Akin H 2020	Giacomet V	Gastrointestinal Symptoms in Severe COVID-19 Children.
Akin H 2020	Cholankeni G	High Prevalence of Concurrent Gastrointestinal Manifestations in Patients With Severe Acute Respiratory Syndrome Coronavirus 2: Early Experience From California.
Akin H 2020	Klopfenstein T	Diarrhea: An under estimated symptom in Corona virus disease 2019.
Akin H 2020	Lin L	Gastrointestinal symptoms of 95 cases with SARS-CoV-2 infection.
Akin H 2020	Kluytmans M	SARS-CoV-2 infection in 86 healthcare workers in two Dutch hospitals in March 2020.
Akin H 2020	Wei X-S	Diarrhea is associated with prolonged symptoms and viral carriage in COVID-19.
Akin H 2020	Cheung KS	Gastrointestinal manifestations of SARS-CoV-2 infection and virus load in fecal samples from the Hong Kong cohort and systematic review and meta-analysis.
Akin H 2020	Wu WS	Investigation and analysis on characteristics of a cluster of COVID-19 associated with exposure in a department store in Tianjin.
Akin H 2020	Feng Z	Early prediction of disease progression in 2019 novel coronavirus pneumonia patients outside Wuhan with CT and clinical characteristics.
Akin H 2020	Wan S	Relationships among lymphocyte subsets, cytokines, and the pulmonary inflammation index in coronavirus (COVID-19) infected patients.
Akin H 2020	Ai J	The cross-sectional study of hospitalized corona virus disease 2019 patients in Xiangyang, Hubei province.
Akin H 2020	Chen N	Epidemiological and clinical characteristics of 99 cases of 2019 novel corona virus pneumonia in Wuhan, China: A descriptive study.
Akin H 2020	Qian GQ	Epidemiologic and Clinical Characteristics of 91 Hospitalized Patients with COVID-19 in Zhejiang, China: A retrospective, multi-centre case series.
Akin H 2020	Zhao XY	Clinical characteristics of patients with 2019 corona virus disease in a non-Wuhan area of Hubei Province, China: A retrospective study.
Akin H 2020	Shi H	Radiological findings from 81 patients with COVID-1 9 pneumonia in Wuhan, China: A descriptive study.
Akin H 2020	Fang X	Clinical characteristics and treatment strategies of 79 patients with COVID-19.
Akin H 2020	Zhao Z	Clinical and laboratory profiles of 75 hospitalized patients with novel corona virus disease 2019 in Hefei, China.
Akin H 2020	Song F	Emerging 2019 novel coronavirus (2019-nCoV) pneumonia.
Akin H 2020	Meng J	Renin-angiotensin system inhibitors improve the clinical outcomes of COVID-19 patients with hypertension.
Akin H 2020	Hu Z	Clinical characteristics of 24 asymptomatic infections with COVID-19 screened among close contacts in Nanjing, China.

Included reviews	Primary studies	Title
Akin H 2020	Liang D	Prevalence and clinical features of 2019 novel coronavirus disease (COVID-19) in the Fever Clinic of a teaching hospital in Beijing: a single-center, retrospective study
Aziz M 2021	Abalo-Lojo	Taste and smell dysfunction in COVID-19 patients
Aziz M 2021	Aggrawal	Clinical features, laboratory characteristics, and outcomes of patients hospitalized with coronavirus disease 2019 (COVID-19): Early report from the United States
Aziz M 2021	Altin F	Olfactory and gustatory abnormalities in COVID-19 cases
Aziz M 2021	Beltrán-Corbellini Á	Acute-onset smell and taste disorders in the context of COVID-19: a pilot multicentre polymerase chain reaction based case-control study
Aziz M 2021	Brandstetter	Symptoms and immunoglobulin development in hospital staff exposed to a SARS-CoV-2 outbreak
Aziz M 2021	Carignan A	Anosmia and dysgeusia associated with SARS-CoV-2 infection: an age-matched case-control study
Aziz M 2021	Chiesa-Estomba (1)	Olfactory and gustatory dysfunctions in COVID-19 first reports of Latin-American ethnic patients
Aziz M 2021	Chiesa-Estomba (2)	Patterns of smell recovery in 751 patients affected by the COVID-19 outbreak
Aziz M 2021	D'Ascanio L	Olfactory dysfunction in COVID-19 patients: prevalence and prognosis for recovering sense of smell
Aziz M 2021	Dawson P	Loss of taste and smell as distinguishing symptoms of COVID-19
Aziz M 2021	Dell'Era V	Smell and taste disorders during COVID-19 outbreak: cross-sectional study on 355 patients
Aziz M 2021	Giacomelli	Self-reported olfactory and taste disorders in patients with severe acute respiratory coronavirus 2 infection: a cross-sectional study
Aziz M 2021	Gorzkowski V	Evolution of olfactory disorders in COVID-19 patients
Aziz M 2021	Guner	COVID-19 experience of the major pandemic response center in the capital: results of the pandemic's first month in Turkey
Aziz M 2021	Haehner A	Predictive value of sudden olfactory loss in the diagnosis of COVID-19
Aziz M 2021	Hintschich	Psychophysical tests reveal impaired olfaction but preserved gustation in COVID-19 patients
Aziz M 2021	Hornus	Anosmia in COVID-19 patients
Aziz M 2021	Izquierdo-Domínguez A	Smell and taste dysfunctions in COVID-19 are associated with younger age in ambulatory settings - a multicenter cross-sectional study
Aziz M 2021	Jalessi M	Frequency and outcome of olfactory impairment and sinonasal involvement in hospitalized patients with COVID-19
Aziz M 2021	Kai Chua	Acute olfactory loss is specific for Covid-19 at the Emergency Department
Aziz M 2021	Kempker	Loss of smell and taste among healthcare personnel screened for coronavirus 2019

**Included reviews**	**Primary studies**	**Title**
Aziz M 2021	Kim GU	Clinical characteristics of asymptomatic and symptomatic patients with mild COVID-19
Aziz M 2021	Klopfenstein T	Features of anosmia in COVID-19
Aziz M 2021	Lechien JR	Olfactory and gustatory dysfunctions as a clinical presentation of mild-to-moderate forms of the coronavirus disease (COVID-19): a multicenter European study
Aziz M 2021	Lechien (2)	Objective olfactory evaluation of self-reported loss of smell in a case series of 86 COVID-19 patients
Aziz M 2021	Lechien JR	Clinical and epidemiological characteristics of 1420 European patients with mild-to-moderate coronavirus disease 2019
Aziz M 2021	Lechien JR	Objective Olfactory Findings in Hospitalized Severe COVID-19 Patients
Aziz M 2021	Lee DJ	Self-reported anosmia and dysgeusia as key symptoms of coronavirus disease 2019
Aziz M 2021	Liang YJ	Neurosensory dysfunction: a diagnostic marker of early COVID-19
Aziz M 2021	Magnavita	Symptoms in health care workers during the COVID-19 epidemic a cross-sectional survey
Aziz M 2021	Mao L	Neurologic manifestations of hospitalized patients with coronavirus disease 2019 in Wuhan, China
Aziz M 2021	Martin-Sanz	Prospective study in 355 patients with suspected COVID-19 infection value of cough, subjective hyposmia, and hypogeusia
Aziz M 2021	Mishra P	Prevalence of new onset anosmia in COVID-19 patients: is the trend different between european and indian population?
Aziz M 2021	Moein ST	Smell dysfunction: a biomarker for COVID-19
Aziz M 2021	Noh	Asymptomatic infection and atypical manifestations of COVID-19: Comparison of viral shedding duration
Aziz M 2021	Paderno A	Olfactory and gustatory outcomes in COVID-19: a prospective evaluation in nonhospitalized subjects
Aziz M 2021	Paderno A	Smell and taste alterations in COVID-19: a cross-sectional analysis of different cohorts
Aziz M 2021	Parente-Arias	Recovery rate and factors associated with smell and taste disruption in patients with coronavirus disease 2019
Aziz M 2021	Patel A	New-onset anosmia and ageusia in adult patients diagnosed with SARS-CoV-2 infection
Aziz M 2021	Petrocelli	Remote psychophysical evaluation of olfactory and gustatory functions in early-stage coronavirus disease 2019 patients: the Bologna experience of 300 cases
Aziz M 2021	Qiu CH	Olfactory and gustatory dysfunction as an early identifier of COVID-19 in adults and children: an international multicenter study
Aziz M 2021	Romero-Sanchez	Neurologic manifestations in hospitalized patients with COVID-19: The ALBACOVID registry
Aziz M 2021	Sakalli E	Ear nose throat-related symptoms with a focus on loss of smell and/or taste in COVID-19 patients

Included reviews	Primary studies	Title
Aziz M 2021	Sayin	Taste and smell impairment in COVID-19: an AAO-HNS anosmia reporting tool-based comparative study
Aziz M 2021	Tostmann	Strong associations and moderate predictive value of early symptoms for SARS-CoV-2 test positivity among healthcare workers, the Netherlands, March 2020
Aziz M 2021	Tsivgoulis	Quantitative evaluation of olfactory dysfunction in hospitalized patients with Coronavirus (COVID-19)
Aziz M 2021	Vaira (1)	Objective evaluation of anosmia and ageusia in COVID-19 patients: Single-center experience on 72 cases
Aziz M 2021	Vaira (2)	Olfactory and gustatory function impairment in COVID-19 patients: Italian objective multicenter-study
Aziz M 2021	Yan CH	Association of chemosensory dysfunction and Covid-19 in patients presenting with influenza-like symptoms
Aziz M 2021	Yan CRH	Self-reported olfactory loss associates with outpatient clinical course in Covid-19
Aziz M 2021	Zayet	Clinical features of COVID-19 and influenza: a comparative study on Nord Franche-Comte cluster
Badal S 2020	Dong Y	Epidemiology of COVID-19 among children in China
Badal S 2020	Peng H	Coronavirus disease 2019 in children: characteristics, antimicrobial treatment, and outcomes
Badal S 2020	Bai K	Clinical analysis of 25 COVID-19 infections in children
Badal S 2020	Du H	Clinical characteristics of 182 pediatric COVID-19 patients with different severities and allergic status
Badal S 2020	Qiu H	Clinical and epidemiological features of 36 children with coronavirus disease 2019 (COVID-19) in Zhejiang, China: an observational cohort study
Badal S 2020	Shen Q	Novel coronavirus infection in children outside of Wuhan, China
Badal S 2020	Song W	Clinical features of pediatric patients with coronavirus disease (COVID-19)
Badal S 2020	Tan YP	Epidemiologic and clinical characteristics of 10 children with coronavirus disease 2019 in Changsha, China
Badal S 2020	Zheng F	Clinical characteristics of children with coronavirus disease 2019 in Hubei, China
Badal S 2020	Zhu L	Clinical characteristics of a case series of children with coronavirus disease 2019
Badal S 2020	Cai J	A case series of children with 2019 novel coronavirus infection: clinical and epidemiological features
Badal S 2020	Lu X	SARS-CoV-2 infection in children
Badal S 2020	Ma H	A single-center, retrospective study of COVID-19 features in children: a descriptive investigation
Badal S 2020	Garazzino S	Multicentre Italian study of SARS-CoV-2 infection in children and adolescents, preliminary data as at 10 April 2020
Badal S 2020	Parri N	Characteristic of COVID-19 infection in pediatric patients: early findings from two Italian Pediatric Research Networks
Badal S 2020	Garcia-Salido A	Children in critical care due to severe acute respiratory syndrome coronavirus 2 infection: experience in a Spanish hospital
Badal S 2020	Foster CE	Coronavirus disease 2019 in children cared for at Texas children’s hospital: initial clinical characteristics and outcomes
Badal S 2020	Zachariah P	Epidemiology, clinical features, and disease severity in patients with coronavirus disease 2019 (COVID-19) in a children’s hospital in New York City, New York

Included reviews	Primary studies	Title
Badal S 2020	Mannheim J	Characteristics of hospitalized pediatric COVID-19 cases - Chicago, Illinois, March – April 2020
Badal S 2020	Shekerdemian LS	Characteristics and outcomes of children with coronavirus disease 2019 (COVID-19) infection admitted to US and Canadian Pediatric Intensive Care Units
Christophers B 2020	Ji LN	Clinical features of pediatric patients with COVID-19: a report of two family cluster cases
Christophers B 2020	Li W	Chest computed tomography in children with COVID-19 respiratory infection
Christophers B 2020	Liu H	Clinical and CT imaging features of the COVID-19 pneumonia: focus on pregnant women and children
Christophers B 2020	Liu M	High-resolution computed tomography manifestations of 5 pediatric patients with 2019 novel coronavirus
Christophers B 2020	Liu W	Clinical characteristics of 19 neonates born to mothers with COVID-19
Christophers B 2020	Lou XX	Three children who recovered from novel coronavirus 2019 pneumonia
Christophers B 2020	Paret M	SARS-CoV-2 infection (COVID-19) in febrile infants without respiratory distress
Christophers B 2020	Parri N	Children with covid-19 in pediatric emergency departments in Italy
Christophers B 2020	Rahimzadeh G	COVID-19 infection in Iranian children: a case series of 9 patients
Christophers B 2020	See KC	COVID-19: four paediatric cases in Malaysia
Christophers B 2020	Shen Q	Novel coronavirus infection in children outside of Wuhan, China
Christophers B 2020	Su L	The different clinical characteristics of corona virus disease cases between children and their families in China - the character of children with COVID-19
Christophers B 2020	Sun D	Clinical features of severe pediatric patients with coronavirus disease 2019 in Wuhan: a single center observational study
Christophers B 2020	Tan YP	Epidemiologic and clinical characteristics of 10 children with coronavirus disease 2019 in Changsha, China
Christophers B 2020	Verdoni L	An outbreak of severe Kawasaki-like disease at the Italian epicentre of the SARS-CoV-2 epidemic: an observational cohort study
Christophers B 2020	Wei M	Novel coronavirus infection in hospitalized infants under 1 year of age in china
Christophers B 2020	Xing Y H	Prolonged viral shedding in feces of pediatric patients with coronavirus disease 2019
Christophers B 2020	Xu R	CT imaging of one extended family cluster of corona virus disease 2019 (COVID-19) including adolescent patients and “silent infection”
Christophers B 2020	Xu Y	Characteristics of pediatric SARS-CoV-2 infection and potential evidence for persistent fecal viral shedding
Christophers B 2020	Zhang T	Detectable SARS-CoV-2 viral RNA in feces of three children during recovery period of COVID-19 pneumonia
Christophers B 2020	Zheng F	Clinical characteristics of children with coronavirus disease 2019 in Hubei, China

Included reviews	Primary studies	Title
Chua TH 2020	Lechien JR	Olfactory and gustatory dysfunctions as a clinical presentation of mild-to-moderate forms of the coronavirus disease (COVID-19): a multicenter European study
Chua TH 2020	Luers	Olfactory and gustatory dysfunction in coronavirus disease 19 (COVID-19)
Chua TH 2020	Mao L	Neurologic manifestations of hospitalized patients with coronavirus disease 2019 in Wuhan, China
Gaythorpe K 2021	Song W	Clinical features of pediatric patients with coronavirus disease (COVID-19)
Gaythorpe K 2021	Bai K	Clinical analysis of 25 novel coronavirus infections in children
Gaythorpe K 2021	Hua CZ	Epidemiological features and viral shedding in children with SARS-CoV-2 infection
Gaythorpe K 2021	Xiong X	A comparison between Chinese children infected with COVID-19 and with SARS
Gaythorpe K 2021	Ma H	A single-center, retrospective study of COVID-19 features in children: a descriptive investigation
Gaythorpe K 2021	Xu Y	Characteristics of pediatric SARS-CoV-2 infection and potential evidence for persistent fecal viral shedding
Gaythorpe K 2021	Lu Y	Clinical characteristics and radiological features of children infected with the 2019 novel coronavirus
Gaythorpe K 2021	Du H	Clinical characteristics of 182 pediatric COVID-19 patients with different severities and allergic status
Gaythorpe K 2021	Dong	Epidemiology of COVID-19 Among Children in China
Gaythorpe K 2021	Russell TW	Estimating the infection and case fatality ratio for coronavirus disease (COVID-19) using age-adjusted data from the outbreak on the Diamond Princess cruise ship
Gaythorpe K 2021	Lu X	SARS-CoV-2 infection in children
Gaythorpe K 2021	Melgosa M	SARS-CoV-2 infection in Spanish children with chronic kidney pathologies
Gaythorpe K 2021	Lu Y	Symptomatic Infection is Associated with Prolonged Duration of Viral Shedding in Mild Coronavirus Disease 2019: A Retrospective Study of 110 Children in Wuhan
Gaythorpe K 2021	Korkmaz M F	The epidemiological and clinical characteristics of 81 children with COVID-19 in a pandemic hospital in Turkey: An observational cohort study
Ghimire S 2021	Guan W	Clinical Characteristics of Coronavirus Disease 2019 in China.
Ghimire S 2021	Young B	Epidemiologic Features and Clinical Course of Patients Infected With SARS-CoV-2 in Singapore
Ghimire S 2021	Pan L	Clinical Characteristics of COVID-19 Patients With Digestive Symptoms in Hubei, China: A Descriptive, Cross-Sectional, Multicenter Study
Ghimire S 2021	Han C	Digestive symptoms in COVID-19 patients with mild disease severity.
Ghimire S 2021	Zhang JJ	Clinical characteristics of 140 patients infected with SARSCoV-2 in Wuhan. China Allergy
Ghimire S 2021	Jin X	Epidemiological, clinical and virological characteristics of 74 cases of coronavirus-infected disease 2019 (COVID-19) with gastrointestinal symptoms
Ghimire S 2021	Nobel Y	Gastrointestinal symptoms and COVID-19: case-control study from the United States
Ghimire S 2021	Zhou Z	Effect of gastrointestinal symptoms on patients infected with COVID-19
Ghimire S 2021	Cheung K	Gastrointestinal Manifestations of SARS-CoV-2 Infection and Virus Load in Fecal Samples From a Hong Kong Cohort: Systematic Review and Meta-analysis
Ghimire S 2021	Luo S	Don’t Overlook Digestive Symptoms in Patients With 2019 Novel Coronavirus Disease (COVID-19)

**Included reviews**	**Primary studies**	**Title**
Ghimire S 2021	Wang D	Clinical characteristics of 138 hospitalized patients with 2019 novel coronavirus-infected pneumonia in Wuhan, China
Ghimire S 2021	Huang C	Clinical features of patients infected with 2019 novel coronavirus in Wuhan, China
Ghimire S 2021	Wang Z	Clinical Features of 69 Cases With Coronavirus Disease 2019 in Wuhan
Ghimire S 2021	Chen N	Epidemiological and clinical characteristics of 99 cases of 2019 novel coronavirus pneumonia in Wuhan, China: a descriptive study
Ghimire S 2021	Wu J	Clinical Characteristics of Imported Cases of Coronavirus Disease 2019 (COVID-19) in Jiangsu Province: A Multicenter Descriptive Study
Ghimire S 2021	Shi H	Radiological findings from 81 patients with COVID-19 pneumonia in Wuhan, China: a descriptive study
Ghimire S 2021	Yang X	Clinical course and outcomes of critically ill patients with SARS-CoV-2 pneumonia in Wuhan, China: a single-centered, retrospective, observational study
Ghimire S 2021	Mo P	Clinical characteristics of refractory COVID-19 pneumonia in Wuhan, China
Ghimire S 2021	Zhou F	Clinical course and risk factors for mortality of adult inpatients with COVID-19 in Wuhan, China: a retrospective cohort study
Ghimire S 2021	Chang D	Epidemiologic and clinical characteristics of novel coronavirus infections involving 13 patients outside Wuhan, China
Ghimire S 2021	Liu K	Clinical characteristics of novel coronavirus cases in tertiary hospitals in Hubei Province
Ghimire S 2021	Cai Q	COVID-19 in a designated infectious diseases hospital outside Hubei Province, China
Ghimire S 2021	Fan Z	Clinical Features of COVID-19-Related Liver Functional Abnormality
Ghimire S 2021	Xu X-W	Clinical findings in a group of patients infected with the 2019 novel coronavirus (SARS-Cov-2) outside of Wuhan, China: retrospective case series
Ghimire S 2021	Zhang B	Clinical characteristics of 82 cases of death from COVID-19
Ghimire S 2021	Huang Y	Clinical characteristics of 36 non-survivors with COVID-19 in Wuhan, China
Ghimire S 2021	Wei X	Clinical and CT features in pediatric patients with COVID-19 infection: different points from adults
Ghimire S 2021	Song F	Emerging 2019 novel coronavirus (2019-NCoV) pneumonia
Ghimire S 2021	Xiao F	Evidence for gastrointestinal infection of SARS-CoV-2
Ghimire S 2021	Cholankeril G	High prevalence of concurrent gastrointestinal manifestations in patients with SARS-CoV-2: early experience from California
Ghimire S 2021	Tabata S	The clinical characteristics of COVID-19: a retrospective analysis of 104 patients from the outbreak on board the Diamond Princess cruise ship in Japan
Ghimire S 2021	Kluytmans M	SARS-CoV-2 infection in 86 healthcare workers in two Dutch hospitals in March 2020
Ghimire S 2021	Hajifathalian K	Gastrointestinal and hepatic manifestations of 2019 novel coronavirus disease in a large cohort of infected patients from New York: clinical implications
Ghimire S 2021	Gritti G	Use of siltuximab in patients with COVID-19 pneumonia requiring ventilatory support
Ghimire S 2021	Goyal	Clinical characteristics of Covid-19 in New York City
Ghimire S 2021	Siso A	Clinical Features of Covid-19 in Barcelona City
Ghimire S 2021	Remes-Troche	Initial gastrointestinal manifestations in patients with SARS-CoV-2 in 112 patients from Veracruz (southeastern Mexico)
Ghimire S 2021	Redd W	Prevalence and characteristics of gastrointestinal symptoms in patients with SARS-CoV-2 infection in the United States: a multicenter cohort study

Included reviews	Primary studies	Title
Hasani H 2020	Chen N	Epidemiological and clinical characteristics of 99 cases of 2019 novel coronavirus pneumonia in Wuhan, China: a descriptive study
Hasani H 2020	Deng SQ	Characteristics of and public health responses to the coronavirus disease 2019 outbreak in China
Hasani H 2020	Huang C	Clinical features of patients infected with 2019 novel coronavirus in Wuhan, China
Hasani H 2020	Guan WJ	Clinical characteristics of coronavirus disease 2019 in China
Hasani H 2020	Huang Y	Clinical characteristics of laboratory confirmed positive cases of SARS-CoV-2 infection in Wuhan, China: A retrospective single center analysis
Hasani H 2020	Liu K	Clinical characteristics of novel coronavirus cases in tertiary hospitals in Hubei Province
Hasani H 2020	Tian S	Characteristics of COVID-19 infection in Beijing
Hasani H 2020	Wang D	Clinical characteristics of 138 hospitalized patients with 2019 novel coronavirus–infected pneumonia in Wuhan, China
Hasani H 2020	Wu J	Clinical characteristics of imported cases of COVID-19 in Jiangsu Province: a multicenter descriptive study
Hasani H 2020	Xu X	Imaging and clinical features of patients with 2019 novel coronavirus SARS-CoV-2
Hasani H 2020	Xu XW	Clinical findings in a group of patients infected with the 2019 novel coronavirus (SARS-Cov-2) outside of Wuhan, China: retrospective case series
Hasani H 2020	Xu YH	Clinical and computed tomographic imaging features of novel coronavirus pneumonia caused by SARS-CoV-2
Hasani H 2020	Yang W	Clinical characteristics and imaging manifestations of the 2019 novel coronavirus disease (COVID-19):A multi-center study in Wenzhou city, Zhejiang, China
Hasani H 2020	Zhang JJ	Clinical characteristics of 140 patients infected with SARS-CoV-2 in Wuhan, China
Hashan MR 2021	Roxby AC	Outbreak investigation of COVID-19 among residents and staff of an independent and assisted living community for older adults in Seattle, Washington
Hashan MR 2021	Arons MM	Presymptomatic SARS-CoV-2 infections and transmission in a skilled nursing facility
Hashan MR 2021	Blain H	Efficacy of a test-retest strategy in residents and health care personnel of a nursing home facing a COVID-19 outbreak
Hashan MR 2021	Sacco G	COVID-19 in seniors: findings and lessons from mass screening in a nursing home
Hashan MR 2021	Hu L	Clinical and epidemiological features of 34 nursing home elderly with coronavirus disease 2019 (COVID-19) in Wuhan, China: an observational cohort study
Hashan MR 2021	Rutten JJ	COVID-19 in nursing homes a study of diagnosis, symptomatology and disease course]
Hashan MR 2021	van den Besselaar JH	A COVID-19 nursing home transmission study: sequence and metadata from weekly testing in an extensive nursing home outbreak
Hashan MR 2021	Kittang BRH	Outbreak of COVID-19 at three nursing homes in Bergen
Hashan MR 2021	Bernabeu-Wittel M	Death risk stratification in elderly patients with covid-19. A comparative cohort study in nursing homes outbreaks
Hashan MR 2021	Graham NSN	SARS-CoV-2 infection, clinical features and outcome of COVID-19 in United Kingdom nursing homes
Hashan MR 2021	Smith E	Testing for SARS-CoV-2 in care home staff and residents in english care homes: a service evaluation
Hashan MR 2021	Patel MC	Asymptomatic SARS-CoV-2 infection and COVID-19 mortality during an outbreak investigation in a skilled nursing facility
Hashan MR 2021	Dora AV	Universal and serial laboratory testing for SARS-CoV-2 at a long-term care skilled nursing facility for veterans - Los Angeles, California, 2020

Included reviews	Primary studies	Title
Hashan MR 2021	Shi SM	Risk factors, presentation, and course of coronavirus disease 2019 in a large, academic long-term care facility
Kim H 2021		
Mutiawati E 2021	Tan JY	A comparative study on the clinical features of COVID-19 with non-SARS-CoV-2 respiratory viral infections.
Mutiawati E 2021	Sahin D	A pandemic center's experience of managing pregnant women with COVID-19 infection in Turkey: A prospective cohort study.
Mutiawati E 2021	Peyrony O	Accuracy of Emergency Department Clinical Findings for Diagnosis of Coronavirus Disease 2019.
Mutiawati E 2021	Beltrán-Corbellini Á	Acute-onset smell and taste disorders in the context of COVID-19: a pilot multicentre polymerase chain reaction based case-control study.
Mutiawati E 2021	Liu JY	Analysis of imported cases of covid-19 in taiwan: A nationwide study.
Mutiawati E 2021	Carignan A	Anosmia and dysgeusia associated with SARS-CoV-2 infection: an age-matched case-control study.
Mutiawati E 2021	Chen A	Are Gastrointestinal Symptoms Specific for Coronavirus 2019 Infection? A Prospective Case-Control Study From the United States.
Mutiawati E 2021	Luigetti M	Assessment of neurological manifestations in hospitalized patients with COVID-19
Mutiawati E 2021	Yan CH	Association of chemosensory dysfunction and COVID-19 in patients presenting with influenza-like symptoms
Mutiawati E 2021	Rojas-Lechuga MJ	Chemosensory dysfunction in COVID-19 out-patients.
Mutiawati E 2021	Neto DB	Chemosensory Dysfunction in COVID-19: Prevalences, Recovery Rates, and Clinical Associations on a Large Brazilian Sample.
Mutiawati E 2021	Durrani M	Chest x-rays findings in covid 19 patients at a university teaching hospital-a descriptive study.
Mutiawati E 2021	Galván Casas C	Classification of the cutaneous manifestations of COVID-19: a rapid prospective nationwide consensus study in Spain with 375 cases.
Mutiawati E 2021	Lechien JR	Clinical and epidemiological characteristics of 1420 European patients with mild-to-moderate coronavirus disease 2019.
Mutiawati E 2021	Kim GU	Clinical characteristics of asymptomatic and symptomatic patients with mild COVID-19.
Mutiawati E 2021	Lapostolle F	Clinical features of 1487 COVID-19 patients with outpatient management in the Greater Paris: the COVID-call study.
Mutiawati E 2021	Corsini Campioli C	Clinical predictors and timing of cessation of viral RNA shedding in patients with COVID-19.
Mutiawati E 2021	Ferreli F	Clinical presentation at the onset of COVID-19 and allergic rhinoconjunctivitis.
Mutiawati E 2021	Rajkumar I	Contemporary Analysis of Olfactory Dysfunction in Mild to Moderate Covid 19 Patients in A Tertiary Health Care Centre.
Mutiawati E 2021	Wang TZ	COVID-19 presenting as anosmia and dysgeusia in New York City emergency departments, March - April, 2020.
Mutiawati E 2021	Cho RHW	COVID-19 Viral Load in the Severity of and Recovery From Olfactory and Gustatory Dysfunction.
Mutiawati E 2021	Kadiane-Oussou NJ	COVID-19: comparative clinical features and outcome in 114 patients with or without pneumonia (Nord Franche-Comte Hospital, France).
Mutiawati E 2021	Nakanishi H	Differential Diagnosis of COVID-19: Importance of Measuring Blood Lymphocytes, Serum Electrolytes, and Olfactory and Taste Functions.
Mutiawati E 2021	Sheng WH	Dysosmia and dysgeusia in patients with COVID-19 in northern Taiwan.
Mutiawati E 2021	Sakalli E	Ear nose throat-related symptoms with a focus on loss of smell and/or taste in COVID-19 patients.

Included reviews	Primary studies	Title
Mutiawati E 2021	Lagi F	Early experience of an infectious and tropical diseases unit during the coronavirus disease (COVID-19) pandemic, Florence, Italy, February to March 2020.
Mutiawati E 2021	Vacchiano V	Early neurological manifestations of hospitalized COVID-19 patients.
Mutiawati E 2021	Amer MA	Early recovery patterns of olfactory disorders in COVID-19 patients; a clinical cohort study.
Mutiawati E 2021	Ombajo LA	Epidemiological And Clinical Characteristics Of Covid-19 Patients In Kenya.
Mutiawati E 2021	Maechler F	Epidemiological and clinical characteristics of SARS-CoV-2 infections at a testing site in Berlin, Germany, March and April 2020—a cross-sectional study.
Mutiawati E 2021	Hussain MH	Epistaxis as a marker for severe acute respiratory syndrome coronavirus-2 status - A prospective study.
Mutiawati E 2021	Shah NN	Evaluation of Olfactory Acuity in Patients with Coronavirus Disease 2019 (COVID-19).
Mutiawati E 2021	Gorzkowski V	Evolution of Olfactory Disorders in COVID-19 Patients.
Mutiawati E 2021	Klopfenstein T	Features of anosmia in COVID-19.
Mutiawati E 2021	Jalessi M	Frequency and outcome of olfactory impairment and sinonasal involvement in hospitalized patients with COVID-19.
Mutiawati E 2021	Liotta EM	Frequent neurologic manifestations and encephalopathy-associated morbidity in Covid-19 patients.
Mutiawati E 2021	Membrilla JA	Headache as a Cardinal Symptom of Coronavirus Disease 2019: A Cross-Sectional Study.
Mutiawati E 2021	Rocha-Filho PAS	Headache associated with COVID-19: Frequency, characteristics and association with anosmia and ageusia.
Mutiawati E 2021	Uygun O	Headache characteristics in COVID-19 pandemic-a survey study.
Mutiawati E 2021	Assaad S	High mortality rate in cancer patients with symptoms of COVID-19 with or without detectable SARS-COV-2 on RT-PCR.
Mutiawati E 2021	Menni C	Loss of smell and taste in combination with other symptoms is a strong predictor of COVID-19 infection.
Mutiawati E 2021	Mohamud MFY	Loss of taste and smell are common clinical characteristics of patients with COVID-19 in somalia: A retrospective double centre study.
Mutiawati E 2021	Dawson P	Loss of Taste and Smell as Distinguishing Symptoms of COVID-19.
Mutiawati E 2021	Çalica Utku A	Main symptoms in patients presenting in the COVID-19 period.
Mutiawati E 2021	Mao L	Neurologic Manifestations of Hospitalized Patients With Coronavirus Disease 2019 in Wuhan, China.
Mutiawati E 2021	Studart-Neto A	Neurological consultations and diagnoses in a large, dedicated COVID-19 university hospital.
Mutiawati E 2021	Pinna P	Neurological manifestations and COVID-19: Experiences from a tertiary care center at the Frontline.
Mutiawati E 2021	Garg R	Neurological symptoms as initial manifestation of Covid-19-An observational study.
Mutiawati E 2021	Liang YJ	Neurosensory dysfunction: A diagnostic marker of early COVID-19.
Mutiawati E 2021	Klopfenstein T	New loss of smell and taste: Uncommon symptoms in COVID-19 patients in Nord Franche-Comte cluster, France.
Mutiawati E 2021	Boscolo-Rizzo P	New onset of loss of smell or taste in household contacts of home-isolated SARS-CoV-2-positive subjects.
Mutiawati E 2021	Patel A	New-onset anosmia and ageusia in adult patients diagnosed with SARS-CoV-2 infection.
Mutiawati E 2021	Vaira LA	Objective evaluation of anosmia and ageusia in COVID-19 patients: Single-center experience on 72 cases.

Included reviews	Primary studies	Title
Mutiawati E 2021	Lechien JR	Objective olfactory findings in hospitalized severe COVID-19 patients
Mutiawati E 2021	Shemer A	Ocular involvement in coronavirus disease 2019 (COVID-19): a clinical and molecular analysis.
Mutiawati E 2021	Altin F	Olfactory and gustatory abnormalities in COVID-19 cases.
Mutiawati E 2021	Qiu CH	Olfactory and Gustatory Dysfunction as an Early Identifier of COVID-19 in Adults and Children: An International Multicenter Study.
Mutiawati E 2021	Ramasamy K	Olfactory and Gustatory Dysfunctions as a Clinical Manifestation of Coronavirus Disease 2019 in a Malaysian Tertiary Center
Mutiawati E 2021	Lechien JR	Olfactory and gustatory dysfunctions as a clinical presentation of mild-to-moderate forms of the coronavirus disease (COVID-19): a multicenter European study.
Mutiawati E 2021	Meini S	Olfactory and gustatory dysfunctions in 100 patients hospitalized for COVID-19: sex differences and recovery time in real-life.
Mutiawati E 2021	Paderno A	Olfactory and Gustatory Outcomes in COVID-19: A Prospective Evaluation in Nonhospitalized Subjects.
Mutiawati E 2021	Speth MM	Olfactory Dysfunction and Sinonasal Symptomatology in COVID-19: Prevalence, Severity, Timing, and Associated Characteristics.
Mutiawati E 2021	D’Ascanio L	Olfactory Dysfunction in COVID-19 Patients: Prevalence and Prognosis for Recovering Sense of Smell.
Mutiawati E 2021	Otte MS	Olfactory dysfunction in patients after recovering from COVID-19
Mutiawati E 2021	Klein H	Onset, duration, and persistence of taste and smell changes and other COVID-19 symptoms: longitudinal study in Israeli patients.
Mutiawati E 2021	Panda S	Otolaryngologic Manifestation and Long-Term Outcome in Mild COVID-19: Experience from a Tertiary Care Centre in India.
Mutiawati E 2021	Özçelik Korkmaz M	Otolaryngological manifestations of hospitalised patients with confirmed COVID-19 infection.
Mutiawati E 2021	Elibol E	Otolaryngological symptoms in COVID-19
Mutiawati E 2021	Lagier JC	Outcomes of 3,737 COVID-19 patients treated with hydroxychloroquine/azithromycin and other regimens in Marseille, France: A retrospective analysis.
Mutiawati E 2021	Uhm JS	Patterns of viral clearance in the natural course of asymptomatic COVID-19: Comparison with symptomatic non-severe COVID-19.
Mutiawati E 2021	Yan CH	Persistent Smell Loss Following Undetectable SARS-CoV-2.
Mutiawati E 2021	López de la Iglesia J	Predictive factors of COVID-19 in patients with negative RT-qPCR.
Mutiawati E 2021	Haehner A	Predictive Value of Sudden Olfactory Loss in the Diagnosis of COVID-19.
Mutiawati E 2021	Fantozzi PJ	Xerostomia, gustatory and olfactory dysfunctions in patients with COVID-19.
Mutiawati E 2021	Moro E	The international European Academy of Neurology survey on neurological symptoms in patients with COVID-19 infection.
Mutiawati E 2021	Joffily L	The close relationship between sudden loss of smell and COVID-19.
Mutiawati E 2021	Bidkar V	Testing Olfactory and Gustatory Dysfunctions among Quarantine COVID-19 Suspects.
Mutiawati E 2021	Cazzolla AP	Taste and Smell Disorders in COVID-19 Patients: Role of Interleukin-6.
Mutiawati E 2021	Salepci E	Symptomatology of COVID-19 from the otorhinolaryngology perspective: a survey of 223 SARS-CoV-2 RNA-positive patients.
Mutiawati E 2021	Freni F	Symptomatology in head and neck district in coronavirus disease (COVID-19): A possible neuroinvasive action of SARS-CoV-2.

Included reviews	Primary studies	Title
Mutiawati E 2021	Foster KJ	Smell loss is a prognostic factor for lower severity of coronavirus disease 2019
Mutiawati E 2021	Moein ST	Smell dysfunction: a biomarker for COVID-19.
Mutiawati E 2021	Lima MA	Smell dysfunction in COVID-19 patients: More than a yes-no question.
Mutiawati E 2021	Vaira LA	Smell and taste recovery in coronavirus disease 2019 patients: A 60-day objective and prospective study.
Mutiawati E 2021	Izquierodo-Dominguez A	Smell and taste dysfunction in covid-19 is associated with younger age in ambulatory settings: A multicenter cross-sectional study.
Mutiawati E 2021	Barón-Sánchez J	Smell and taste disorders in Spanish patients with mild COVID-19.
Mutiawati E 2021	Dell'Era V	Smell and taste disorders during COVID-19 outbreak: Cross-sectional study on 355 patients.
Mutiawati E 2021	Paderno A	Smell and taste alterations in COVID-19: a cross-sectional analysis of different cohorts.
Mutiawati E 2021	Alshami A	Silent disease and loss of taste and smell are common manifestations of SARS-COV-2 infection in a quarantine facility: Saudi Arabia.
Mutiawati E 2021	Konstantinidis I	Short-term follow-up of self-isolated covid-19 patients with smell and taste dysfunction in Greece: Two phenotypes of recovery.
Mutiawati E 2021	DeBiasi RL	Severe Coronavirus Disease-2019 in Children and Young Adults in the Washington, DC, Metropolitan Region
Mutiawati E 2021	Song J	Self-reported taste and smell disorders in patients with COVID-19: distinct features in China.
Mutiawati E 2021	Yan CRH	Self-reported olfactory loss associates with outpatient clinical course in COVID-19
Mutiawati E 2021	Lee DJ	Self-reported anosmia and dysgeusia as key symptoms of coronavirus disease 2019
Mutiawati E 2021	Karni N	Self-rated smell ability enables highly specific predictors of COVID-19 status: a case control study in Israel.
Mutiawati E 2021	Avcl H	Relationship between anosmia and hospitalisation in patients with coronavirus disease 2019: An otolaryngological perspective.
Mutiawati E 2021	Mishra P	Prevalence of New Onset Anosmia in COVID-19 Patients: Is The Trend Different Between European and Indian Population?
Mutiawati E 2021	Nouchi A	Prevalence of hyposmia and hypogeusia in 390 COVID-19 hospitalized patients and outpatients: a cross-sectional study.
Mutiawati E 2021	Vivek Kumar P	Prevalence Of Anosmia And Dysgeusia In Patients Of COVID-19 In A Dedicated Covid Hospital.
Mutiawati E 2021	Al-Ani RM	Prevalence of Anosmia and Ageusia in Patients with COVID-19 at a Primary Health Center, Doha, Qatar.
Mutiawati E 2021	Moein ST	Prevalence and reversibility of smell dysfunction measured psychophysically in a cohort of COVID-19 patients.
Mutiawati E 2021	Lv H	Prevalence and recovery time of olfactory and gustatory dysfunction in hospitalized patients with COVID-19 in Wuhan.
Mutiawati E 2021	Chary E	Prevalence and Recovery From Olfactory and Gustatory Dysfunctions in Covid-19 Infection: A Prospective Multicenter Study.
Mutiawati E 2021	Lee Y	Prevalence and Duration of Acute Loss of Smell or Taste in COVID-19 Patients.
Mutiawati E 2021	Redd WD	Prevalence and Characteristics of Gastrointestinal Symptoms in Patients With Severe Acute Respiratory Syndrome Coronavirus 2 Infection in the United States: A Multicenter Cohort Study.
Mutiawati E 2021	Pinato DJ	Presenting features and early mortality from SARS-CoV-2 infection in cancer patients during the initial stage of the COVID-19 pandemic in Europe
Mutiawati E 2021	O'Keefe JB	Predictors of disease duration and symptom course of outpatients with acute covid-19: a retrospective cohort study.
Mutiawati E 2020	Nakagawara K	Acute onset olfactory/taste disorders are associated with a high viral burden in mild or asymptomatic SARS-CoV-2 infections

Included reviews	Primary studies	Title
Mutiawati E 2020	Luigetii M	Assessment of neurological manifestations in hospitalized patients with COVID-19
Mutiawati E 2020	Liu Z	Association between Initial Chest CT or Clinical Features and Clinical Course in Patients with Coronavirus Disease 2019 Pneumonia
Mutiawati E 2020	Yan CH	Association of chemosensory dysfunction and COVID-19 in patients presenting with influenza-like symptoms
Mutiawati E 2020	Devaux CA	New insights on the antiviral effects of chloroquine against coronavirus: what to expect for COVID-19?
Mutiawati E 2020	dÉttorre G	Challenges in the Management of SARS-CoV2 Infection: The Role of Oral Bacteriotherapy as Complementary Therapeutic Strategy to Avoid the Progression of COVID-19
Mutiawati E 2020	Tian S	Characteristics of COVID-19 infection in Beijing
Mutiawati E 2020	Ellington S	Characteristics of Women of Reproductive Age with Laboratory-Confirmed SARS-CoV-2 Infection by Pregnancy Status - United States, January 22-June 7,
Mutiawati E 2020	Ghweil AA	Characteristics, Outcomes and Indicators of Severity for COVID-19 Among Sample of ESNA Quarantine Hospital's Patients, Egypt: A Retrospective Study
Mutiawati E 2020	Bhandari S	Characteristics, Treatment Outcomes and Role of Hydroxychloroquine among 522 COVID-19 hospitalized patients in Jaipur City: An Epidemio-Clinical Study
Mutiawati E 2020	Lei P	Clinical and computed tomographic (CT) images characteristics in the patients with COVID-19 infection: What should radiologists need to know?
Mutiawati E 2020	Xu YH	Clinical and computed tomographic imaging features of novel coronavirus pneumonia caused by SARS-CoV-2
Mutiawati E 2020	Gupta N	Clinical and epidemiologic profile of the initial COVID-19 patients at a tertiary care centre in India
Mutiawati E 2020	Qiu H	Clinical and epidemiological features of 36 children with coronavirus disease 2019 (COVID-19) in Zhejiang, China: an observational cohort study
Mutiawati E 2020	Chen G	Clinical and immunological features of severe and moderate coronavirus disease 2019
Mutiawati E 2020	Wang Y	Clinical and radiological characteristics of COVID-19: a multicentre, retrospective, observational study
Mutiawati E 2020	Tian S	Clinical Characteristics and Reasons for Differences in Duration From Symptom Onset to Release From Quarantine Among Patients With COVID-19 in Liaocheng, China
Mutiawati E 2020	Kim G	Clinical characteristics of asymptomatic and symptomatic patients with mild COVID-19
Mutiawati E 2020	Du W	Clinical characteristics of COVID-19 in children compared with adults in Shandong Province, China
Mutiawati E 2020	Liu K	Clinical characteristics of novel coronavirus cases in tertiary hospitals in Hubei Province
Mutiawati E 2020	He R	The clinical course and its correlated immune status in COVID-19 pneumonia
Mutiawati E 2020	Zhang G	Clinical features and short-term outcomes of 221 patients with COVID-19 in Wuhan, China
Mutiawati E 2020	Du Y	Clinical Features of 85 Fatal Cases of COVID-19 from Wuhan A Retrospective Observational Study
Mutiawati E 2020	Lapostolle F	Clinical features of 1487 COVID-19 patients with outpatient management in the Greater Paris: the COVID-call study
Mutiawati E 2020	Zayet S	Clinical features of COVID-19 and influenza: a comparative study on Nord Franche-Comte cluster
Mutiawati E 2020	Huang C	Clinical features of patients infected with 2019 novel coronavirus in Wuhan, China
Mutiawati E 2020	XU XW	Clinical findings in a group of patients infected with the 2019 novel coronavirus (SARS-Cov-2) outside of Wuhan, China: retrospective case series
Mutiawati E 2020	Huang R	Clinical findings of patients with coronavirus disease 2019 in Jiangsu province, China: A retrospective, multi-center study

Included reviews	Primary studies	Title
Mutiawati E 2020	Corsini C	Clinical predictors and timing of cessation of viral RNA shedding in patients with COVID-19
Mutiawati E 2020	Liu Z	Clinical Time Features and Chest Imaging of 85 Patients With COVID-19 in Zhuhai, China
Mutiawati E 2020	Lian JS	Comparison of epidemiological and clinical characteristics of COVID-19 patients with and without Wuhan exposure history in Zhejiang Province, China
Mutiawati E 2020	Samrah SM	COVID-19 outbreak in Jordan: Epidemiological features, clinical characteristics, and laboratory findings
Mutiawati E 2020	He JL	Diagnostic performance between CT and initial real-time RT-PCR for clinically suspected 2019 coronavirus disease (COVID-19) patients outside Wuhan, China
Mutiawati E 2020	Miyamae Y	Duration of viral shedding in asymptomatic or mild cases of novel coronavirus disease 2019 (COVID-19) from a cruise ship: A single-hospital experience in Tokyo, Japan
Mutiawati E 2020	Ding X	Dynamic profile and clinical implications of hematological parameters in hospitalized patients with coronavirus disease 2019
Mutiawati E 2020	Han R	Early Clinical and CT Manifestations of Coronavirus Disease 2019 (COVID-19) Pneumonia
Mutiawati E 2020	Vacchiano V	Early neurological manifestations of hospitalized COVID-19 patients
Mutiawati E 2020	Dong X	Eleven faces of coronavirus disease 2019
Mutiawati E 2020	Song F	Emerging 2019 Novel Coronavirus (2019-nCoV) Pneumonia
Mutiawati E 2020	Chen N	Epidemiological and clinical characteristics of 99 cases of 2019 novel coronavirus pneumonia in Wuhan, China: a descriptive study
Mutiawati E 2020	Chen P	Epidemiological and clinical characteristics of 136 cases of COVID-19 in main district of Chongqing
Mutiawati E 2020	Lee JY	Epidemiological and clinical characteristics of coronavirus disease 2019 in Daegu, South Korea
Mutiawati E 2020	Zhong ZF	Epidemiological and clinical characteristics of COVID-19 patients in Hengyang, Hunan Province, China
Mutiawati E 2020	Lu R	Epidemiological and clinical characteristics of COVID-19 patients in Nantong, China
Mutiawati E 2020	Liu L	Epidemiological and Clinical Characteristics of Patients With Coronavirus Disease-2019 in Shiyan City, China
Mutiawati E 2020	Wang R	Epidemiological and clinical features of 125 Hospitalized Patients with COVID-19 in Fuyang, Anhui, China
Mutiawati E 2020	Jin X	Epidemiological, clinical and virological characteristics of 74 cases of coronavirus-infected disease 2019 (COVID-19) with gastrointestinal symptoms
Mutiawati E 2020	Lam HY	The epidemiology of COVID-19 cases and the successful containment strategy in Hong Kong-January to May 2020
Mutiawati E 2020	Hu X	Factors associated with negative conversion of viral RNA in patients hospitalized with COVID-19
Mutiawati E 2020	Trigo J	Factors associated with the presence of headache in hospitalized COVID-19 patients and impact on prognosis: a retrospective cohort study
Mutiawati E 2020	Garcia-Azorin D	Frequency and Type of Red Flags in Patients With Covid-19 and Headache: A Series of 104 Hospitalized Patients
Mutiawati E 2020	Poncet-Magemont L	High Prevalence of Headaches During Covid-19 Infection: A Retrospective Cohort Study
Mutiawati E 2020	Lu X	High Resolution CT Imaging Dynamic Follow-Up Study of Novel Coronavirus Pneumonia
Mutiawati E 2020	Li J	Identification of Symptoms Prognostic of COVID-19 Severity: Multivariate Data Analysis of a Case Series in Henan Province
Mutiawati E 2020	Yin S	The implications of preliminary screening and diagnosis: Clinical characteristics of 33 mild patients with SARS-CoV-2 infection in Hunan, China

Included reviews	Primary studies	Title
Mutiawati E 2020	Kosugi EM	Incomplete and late recovery of sudden olfactory dysfunction in COVID-19
Mutiawati E 2020	Shao F	In-hospital cardiac arrest outcomes among patients with COVID-19 pneumonia in Wuhan, China
Mutiawati E 2020	Luo Y	Investigation of COVID-19-related symptoms based on factor analysis
Mutiawati E 2020	Farah Yusuf M	Loss of Taste and Smell are Common Clinical Characteristics of Patients with COVID-19 in Somalia: A Retrospective Double Centre Study
Mutiawati E 2020	Musolino AM	Lung Ultrasound in Children with COVID-19: Preliminary Findings
Mutiawati E 2020	Calika Utku A	Main symptoms in patients presenting in the COVID-19 period
Mutiawati E 2020	Rokohl C	More than loss of taste and smell: burning watering eyes in coronavirus disease 2019
Mutiawati E 2020	Kremer S	Neurologic and neuroimaging findings in patients with COVID-19: A retrospective multicenter study
Mutiawati E 2020	Mao L	Neurologic Manifestations of Hospitalized Patients With Coronavirus Disease 2019 in Wuhan, China
Mutiawati E 2020	Bergquist SH	Non-hospitalized Adults with COVID-19 Differ Noticeably from Hospitalized Adults in Their Demographic, Clinical, and Social Characteristics
Mutiawati E 2020	Vaira LA	Objective evaluation of anosmia and ageusia in COVID-19 patients: Single-center experience on 72 cases
Mutiawati E 2020	DÁscanio L	Olfactory Dysfunction in COVID-19 Patients: Prevalence and Prognosis for Recovering Sense of Smell
Mutiawati E 2020	Liu L	Optimizing screening strategies for coronavirus disease 2019: A study from Middle China
Mutiawati E 2020	Karadas O	A prospective clinical study of detailed neurological manifestations in patients with COVID-19
Mutiawati E 2020	Huang L	Rapid asymptomatic transmission of COVID-19 during the incubation period demonstrating strong infectivity in a cluster of youngsters aged 16-23 years outside Wuhan and characteristics of young patients with COVID-19: A prospective contact-tracing study
Mutiawati E 2020	Wu Y	Relationship between ABO blood group distribution and clinical characteristics in patients with COVID-19
Mutiawati E 2020	He S	Relationship between chest CT manifestations and immune response in COVID-19 patients
Mutiawati E 2020	Zhao J	A Retrospective Analysis of the Clinical and Epidemiological Characteristics of COVID-19 Patients in Henan Provincial People's Hospital, Zhengzhou, China
Mutiawati E 2020	Yao Q	A retrospective study of risk factors for severe acute respiratory syndrome coronavirus 2 infections in hospitalized adult patients
Mutiawati E 2020	Zhang J	Risk factors for disease severity, unimprovement, and mortality in COVID-19 patients in Wuhan, China
Mutiawati E 2020	Palaiodimos L	Severe obesity, increasing age and male sex are independently associated with worse in-hospital outcomes, and higher in-hospital mortality, in a cohort of patients with COVID-19 in the Bronx, New York
Mutiawati E 2020	Gelardi M	Smell and taste dysfunction during the COVID-19 outbreak: a preliminary report
Mutiawati E 2020	Hou H	Using IL-2R/lymphocytes for predicting the clinical progression of patients with COVID-19
Syangtan G; 2021	Bagget TP	Prevalence of SARS-CoV-2 infection in residents of a large homeless shelter in Boston.
Syangtan G; 2021	Dong Y	Epidemiology of COVID-19 among children in China.
Syangtan G; 2021	Hua C Z	Clinical characteristics of 24 asymptomatic infections with COVID-19 screened among close contacts in Nanjing, China.
Syangtan G; 2021	Kimball A	Asymptomatic and presymptomatic SARS-CoV-2 infections in residents of a long-term care skilled nursing facility - King County, Washington, March 2020.

Included reviews	Primary studies	Title
Syangtan G; 2021	Lu X	SARS-CoV-2 infection in children.
Syangtan G; 2021	Meng H	CT imaging and clinical course of asymptomatic cases with COVID-19 pneumonia at admission in Wuhan, China.
Syangtan G; 2021	Mizumoto K	Estimating the asymptomatic proportion of coronavirus disease 2019 (COVID-19) cases on board the Diamond Princess cruise ship, Yokohama, Japan, 2020.
Syangtan G; 2021	NisiuraH	Estimation of the asymptomatic ratio of novel coronavirus infections (COVID-19).
Syangtan G; 2021	Pan Y	Epidemiological and clinical characteristics of 26 asymptomatic SARS-CoV-2 carriers.
Syangtan G; 2021	Qiu H	Clinical and epidemiological features of 36 children with coronavirus disease 2019 (COVID-19) in Zhejiang, China: an observational cohort study.
Syangtan G; 2021	Song H	A considerable proportion of individuals with asymptomatic SARS-CoV-2 infection in Tibetan population.
Syangtan G; 2021	Sutton D	Universal screening for SARS-CoV-2 in women admitted for delivery.
Syangtan G; 2021	Tao Y	High incidence of asymptomatic SARS-CoV-2 infection, Chongqing, China.
Syangtan G; 2021	Tian S	Characteristics of COVID-19 infection in Beijing
Syangtan G; 2021	Wang Y	Clinical outcomes in 55 patients with severe acute respiratory syndrome coronavirus 2 who were asymptomatic at hospital admission in Shenzhen, China.
Syangtan G; 2021	Zhou X	Follow-up of asymptomatic patients with SARS-CoV-2 infection.

Table S6 The number of overlapping primary studies between reviews

	Syangtan G 2021	bMutiawati E 2021	aMutiawati E 2021	Hashan MR 2021	Hasani H 2020	Ghimire S 2021	Gaythorpe K 2021	Chua TH 2020	Christophers B 2020	Badal S 2020	Aziz M 2021	Akin H 2020
Akin H 2020	1	4	2	0	5	14	1	1	0	2	1	76
Aziz M 2021	0	3	27	0	0	0	0	2	0	0	51	1
Badal S 2020	3	1	0	0	0	0	6	0	3	20	0	2
Christophers B 2020	0	0	0	0	0	0	1	0	21	3	0	0
Chua TH 2020	0	1	2	0	0	0	0	3	0	0	2	1
Gaythorpe K 2021	3	0	0	0	0	0	14	0	1	6	0	1
Ghimire S 2021	0	6	1	0	8	38	0	0	0	0	0	14
Hasani H 2020	1	6	0	0	14	8	0	0	0	0	0	5
Hashan MR 2021	0	0	0	14	0	0	0	0	0	0	0	0
aMutiawati E 2021	0	11	107	0	0	1	0	2	0	0	27	2
bMutiawati E 2021	0	78	11	0	6	6	0	1	0	1	3	4
Syangtan G 2021	16	0	0	0	1	0	3	0	0	3	0	1

Appendix 2.3: Figures for sensitivity analysis looking at latest reviews for all ages, children, and adults

Figure S1. Prevalence of each symptom in all ages, extracted from the latest review only

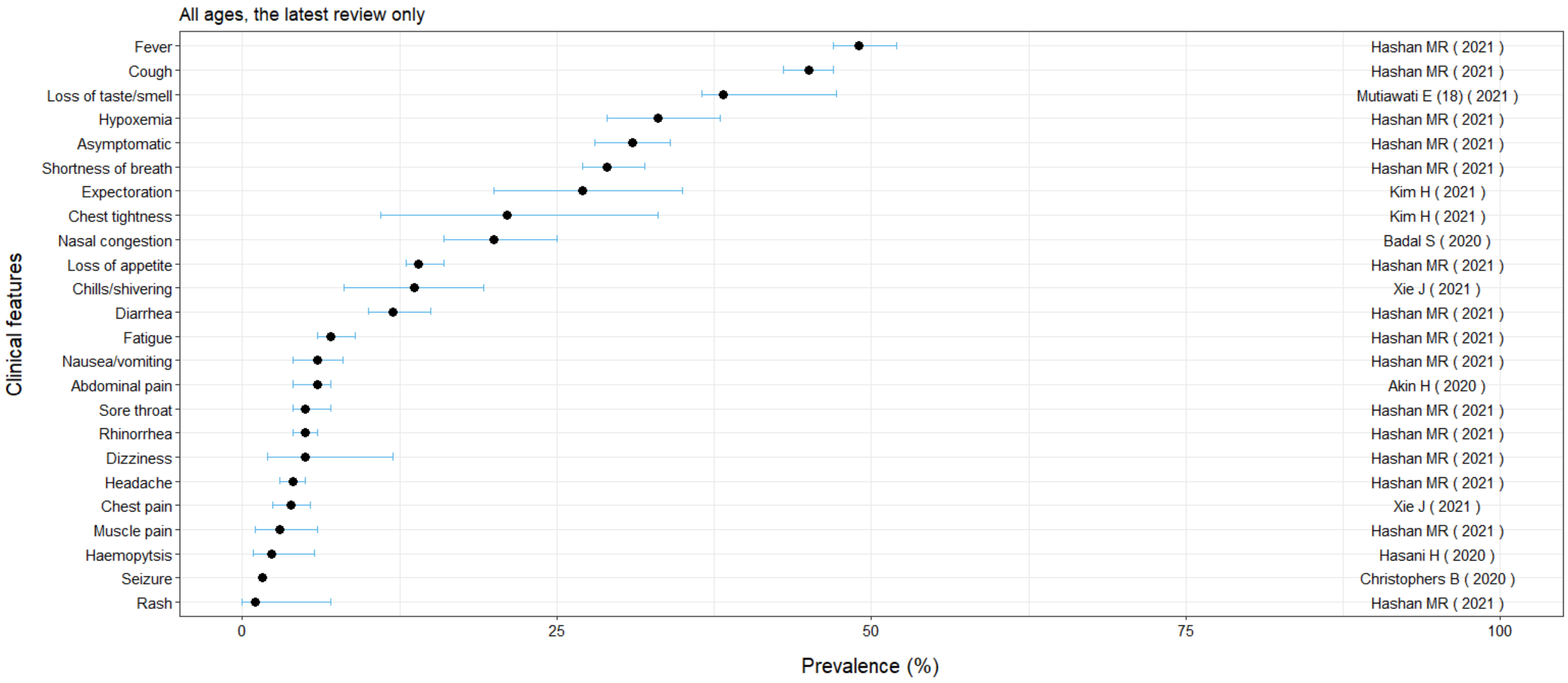

Figure S2. Prevalence of each symptom in children, extracted from the latest review only

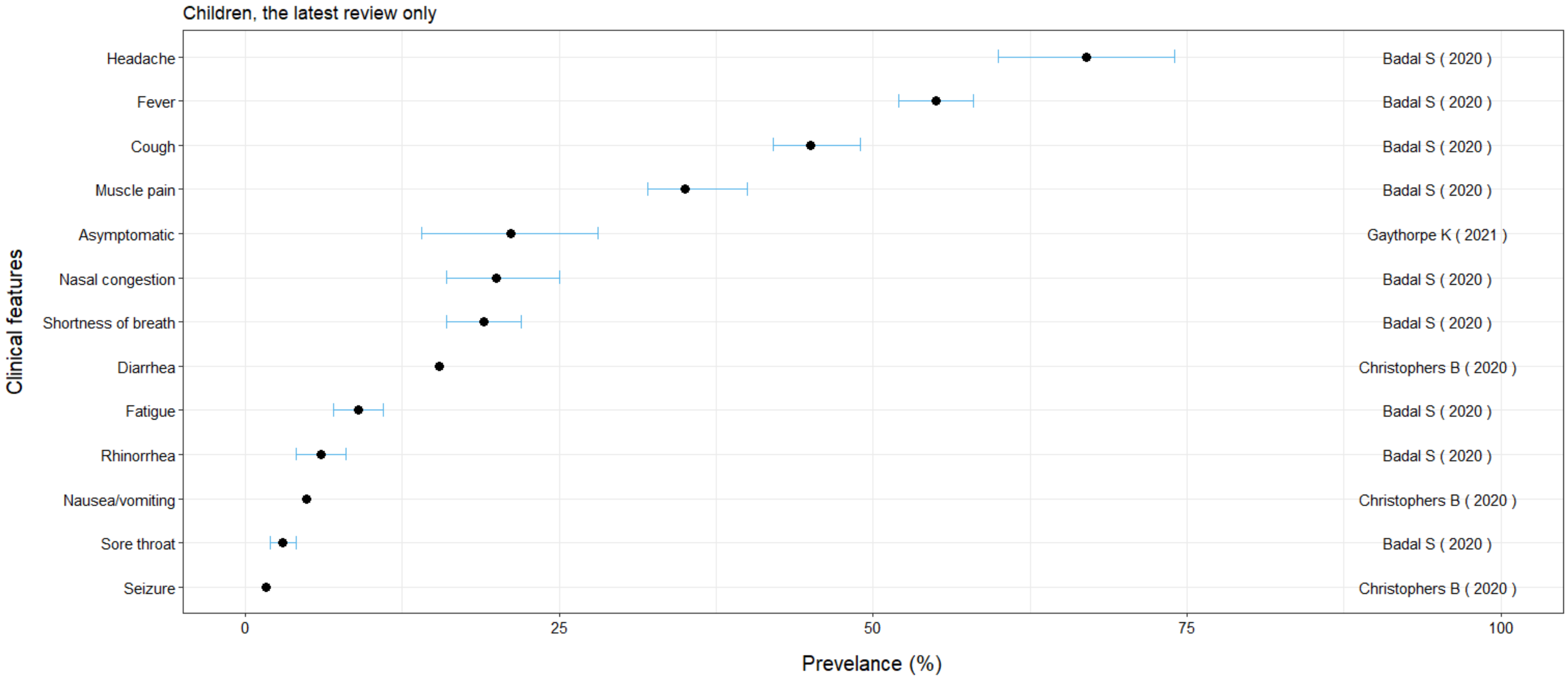

Figure S3. Prevalence of each symptom in adults, extracted from the latest review only

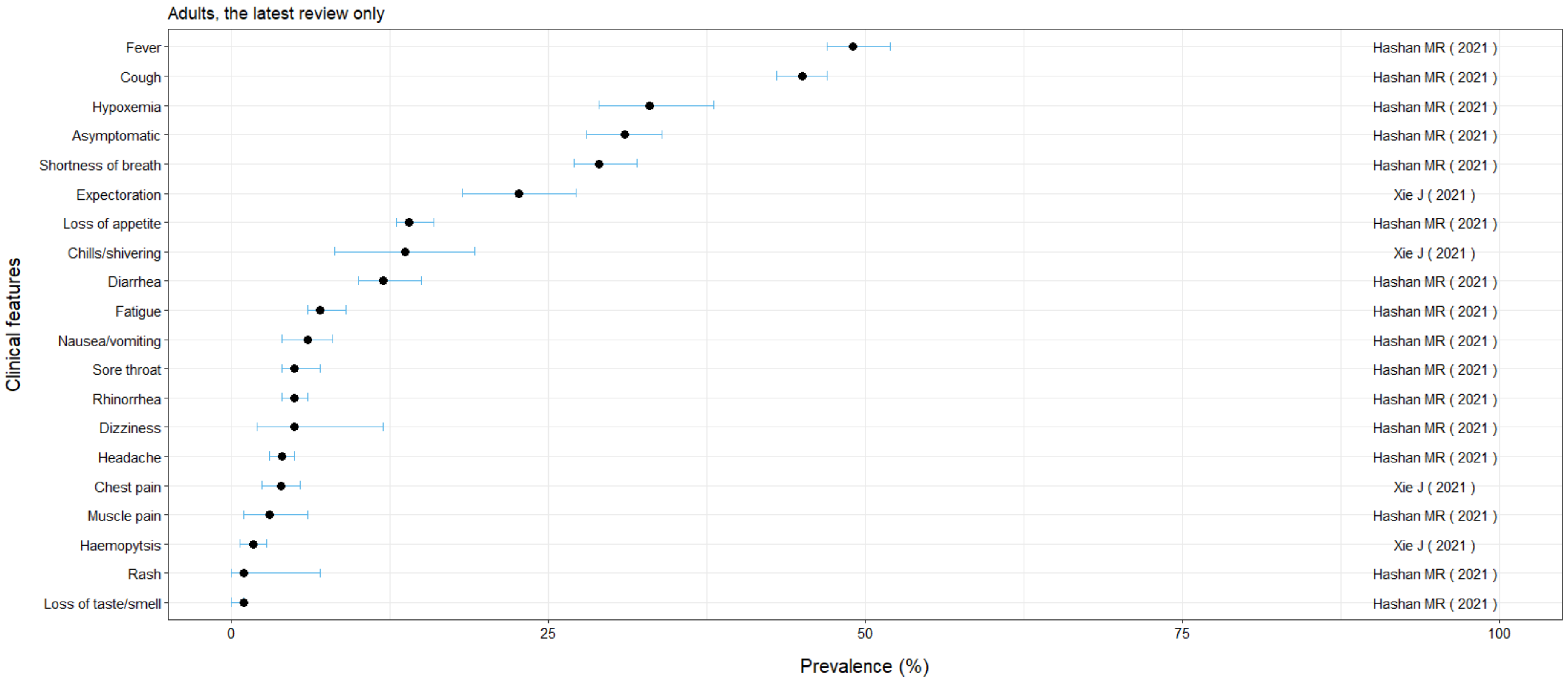

Figure S4. Prevalence of each symptom in all ages, extracted from reviews having at least five clinical features only (n= 7)

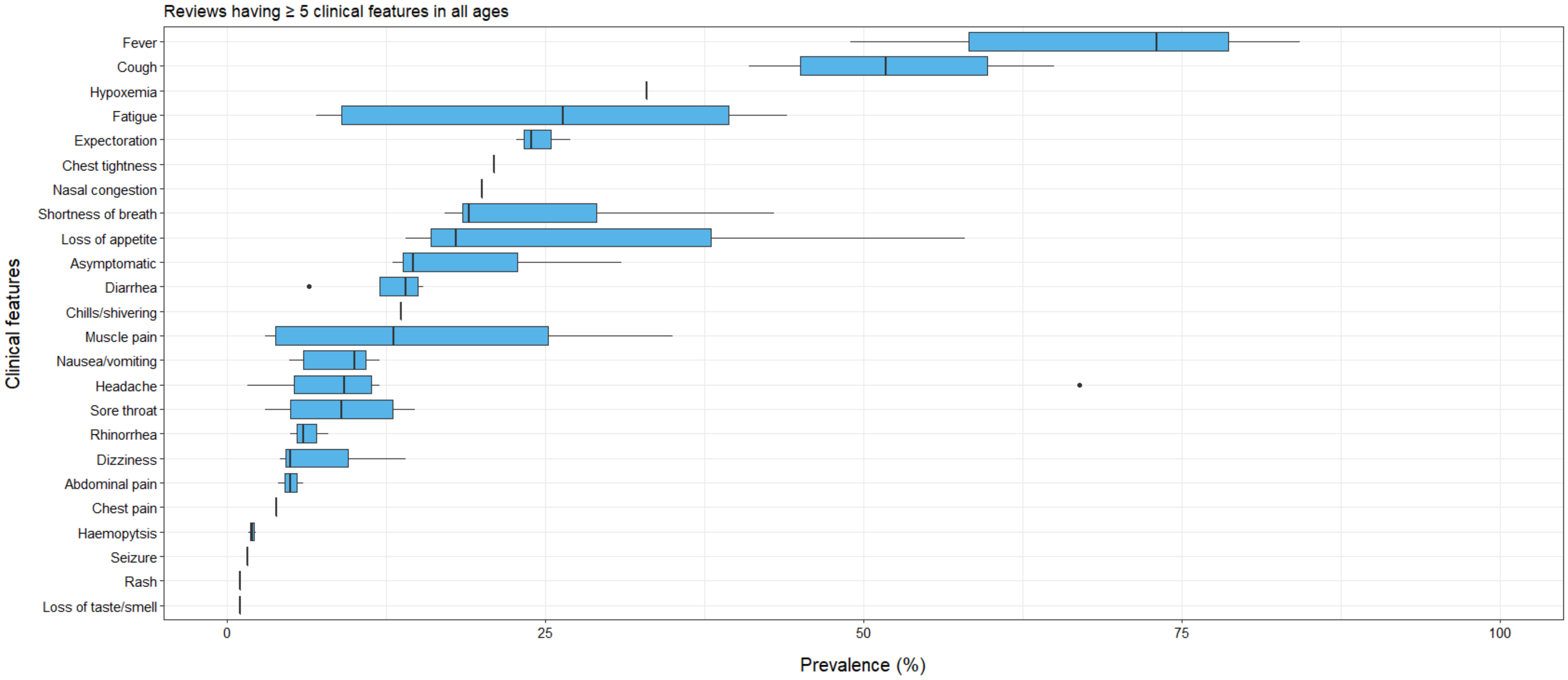

Figure S5. Prevalence of each symptom in children, extracted from reviews having at least five clinical features only (n= 2)

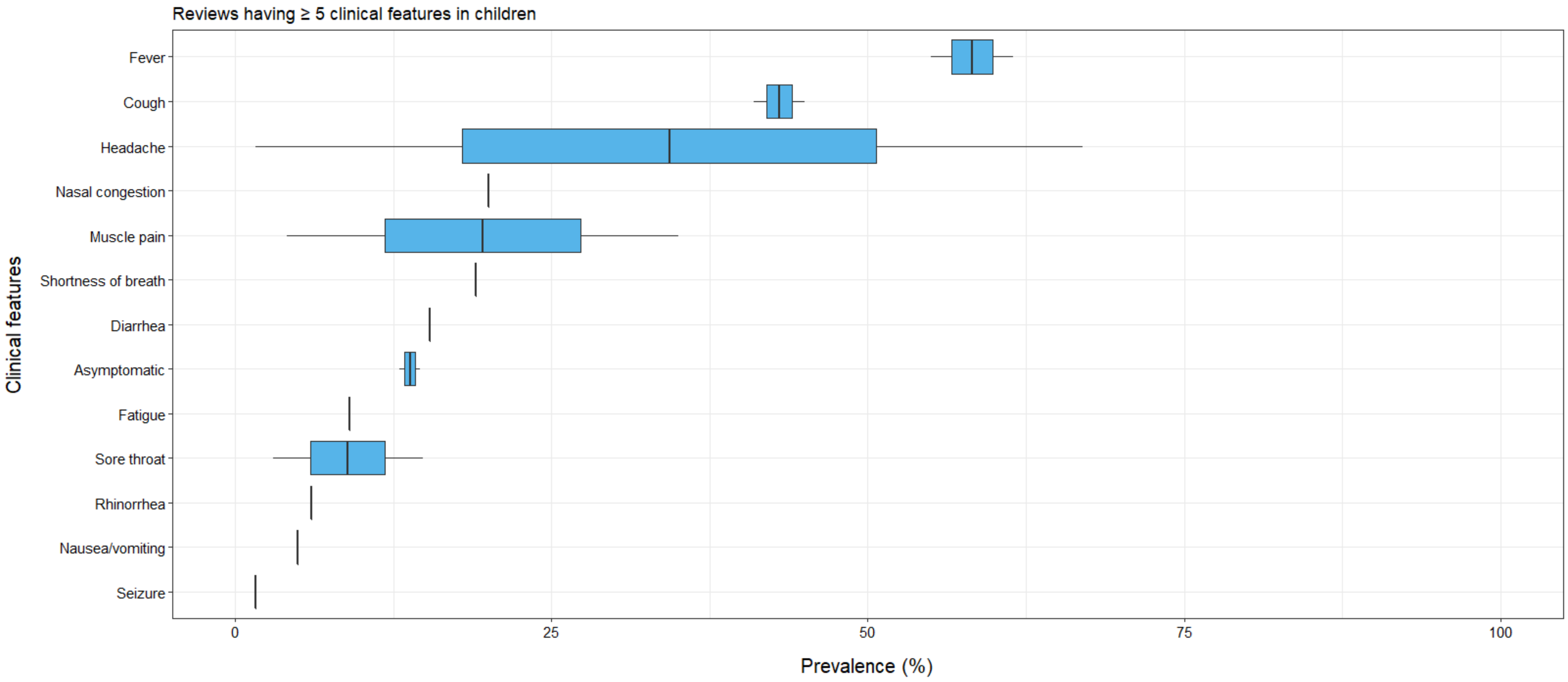

Figure S6. Prevalence of each symptom in adults, extracted from reviews having at least five clinical features only (n= 2)

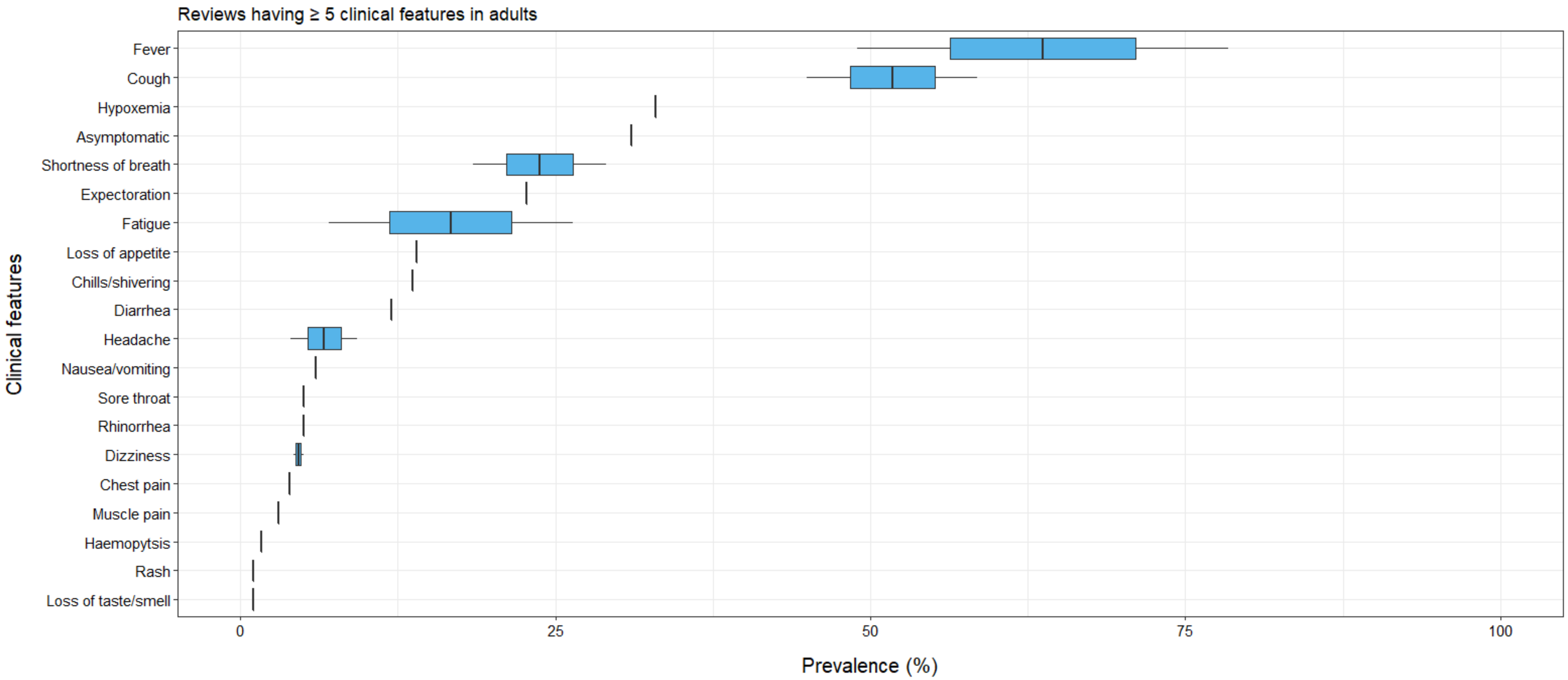

Supplement: Online Supplementary Document [file jogh-12-05012-s001.pdf]
